# Temporal trends analysis of human brucellosis incidence in mainland China from 2004 to 2018

**DOI:** 10.1038/s41598-018-33165-9

**Published:** 2018-10-26

**Authors:** Yongbin Wang, Chunjie Xu, Shengkui Zhang, Zhende Wang, Ying Zhu, Juxiang Yuan

**Affiliations:** 1 School of Public Health, North China University of Science and Technology, Tangshan, Hebei Province P. R. China; 2 School of Public Health, Capital Medical University, Beijing, 100069 P. R. China

## Abstract

With the re-emergence of brucellosis in mainland China since the mid-1990s, an increasing threat to public health tends to become even more violent, advanced warning plays a pivotal role in the control of brucellosis. However, a model integrating the autoregressive integrated moving average (ARIMA) with Error-Trend-Seasonal (ETS) methods remains unexplored in the epidemiological prediction. The hybrid ARIMA-ETS model based on discrete wavelet transform was hence constructed to assess the epidemics of human brucellosis from January 2004 to February 2018 in mainland China. The preferred hybrid model including the best-performing ARIMA method for approximation-forecasting and the best-fitting ETS approach for detail-forecasting is evidently superior to the standard ARIMA and ETS techniques in both three in-sample simulating and out-of-sample forecasting horizons in terms of the minimum performance indices of the root mean square error, mean absolute error, mean error rate and mean absolute percentage error. Whereafter, an ahead prediction from March to December in 2018 displays a dropping trend compared to the preceding years. But being still present, in various trends, in the present or future. This hybrid model can be highlighted in predicting the temporal trends of human brucellosis, which may act as the potential for far-reaching implications for prevention and control of this disease.

## Introduction

Brucellosis is a globally infectious allergic zoonosis caused by bacteria of Brucella spp., the disease can predominantly be transmitted to humans, among whom some special occupational exposures remain to be at potential risk, particularly for farmers, herdsmen, slaughterhouse workers and veterinary workers^1–3^, through contact with the infected animals, especially like cattle, sheep, pigs, dogs, camels and deer, together with consumption of contaminated products, which further spurs the acute and chronic diseases in humans^4,5^. The past decade has witnessed a drastic evolution of brucellosis in the world because of the global varying sanitary, socio-economic and political aspects, along with the rapid development of tourism among nations and regions^3,6^. At present, brucellosis is distributed in more than 160 countries and regions around the world, among which declaring the eradication of brucellosis incidence are only followed by 17 industrialized countries or regions^7^. There still are more than incident 500,000 cases of human brucellosis with billions of dollars in economic losses annually around the world, and even the annual incidence exceeds 10 per million population in some endemic areas^7–9^. More importantly, according to the report conducted by the World Health Organization (WHO) that the actual incidence cases are 10–25 times as many as the notified^10^, i.e., the fact that influence cast by brucellosis on national health and losses to economy is more serious than what we have observed.

With the emerging and reemerging foci occurrence of brucellosis, especially in the developing countries of Asia^8,11,12^, China is one of the quintessential countries, where the morbidity of brucellosis has tardily been on the rise since the middle and late 1990s^13^, while such increasing epidemic has become more pronounced with the acceleration of approximately annual 10%^14^ over the past decade, ranking top 10 of the total cases in class A and B national notifiable infectious diseases reported in mainland China^4^. Currently, the epidemic areas of brucellosis are mainly occurred in the northern regions of China^5^, the sporadic areas primarily in the southern regions of China^6^. However, with the growing development of China’s livestock production, together with the bulk of infected cases get undiagnosed and untreated due to the various vague clinical symptoms and signs^10,11,15^, the affected regions have gradually expanded from north to south and the occurrence of outbreaks becomes increasing popular in China in present-day society^3,16^. Brucellosis has not only posed a threat to the national health but exerted an impact on the development of animal husbandry as well, and having still been considered as a serious public health issue that fails to be ignored on the Chinese mainland^11,17^. Forecasting is invariably seen as an indispensible part of the prevention and control of diseases. Therefore, it is imperative that prediction models with robust accuracy and precision should be erected for the sake of detecting and analyzing the temporal trends, which, if any, is of significant practical implications for reasonably making resource utilization and preventing the morbidity of brucellosis-induced diseases.

At present, the methodologies utilized to predict the incidence of infectious diseases are chiefly linear models, including autoregressive integrated moving average (ARIMA) model, residual autoregressive model, exponential smoothing (ES) and autoregressive distributed lag, and nonlinear models, including prevailingly artificial neural network models, together with their combinations^18–23^. Generally, components of infectious diseases consist of secular trend, periodicity, seasonality and random fluctuation^24^. To improve the simulation and prediction capacity, the optimal prediction method is expected to take full advantage of these diverse data components^25^. Currently, the discrete wavelet transform (DWT) is widely applied in many fields of science and engineering for filtering and preliminary manipulation of raw data to extract fulfilling information included, which further allows for more accurate forecasting and analysis of the current and emerging trends of time series^25–28^. And the Error-Trend-Seasonal (ETS) model that embeds the classical ES models (e.g., Holt and Holt–Winters additive and multiplicative approaches) in a dynamic nonlinear method framework can well recognize seasonal and error patterns in various additive and multiplicative combinations^29,30^. Accordingly, given traits of the human brucellosis incidence time series, our study employed the coif1 method of one-dimensional DWT to block this series into the approximate and detailed scale parts^25^, then the informative implications of approximation were excavated with an ARIMA model, the detailed scale part was mined with an ETS model. Thus, the combined ARIMA-ETS model can realize the goal of absorbing the essence and neglecting the drawbacks of single model for first forecasting the human brucellosis incidence on the Chinese mainland.

## Results

### General characteristics

**Figure 1:**
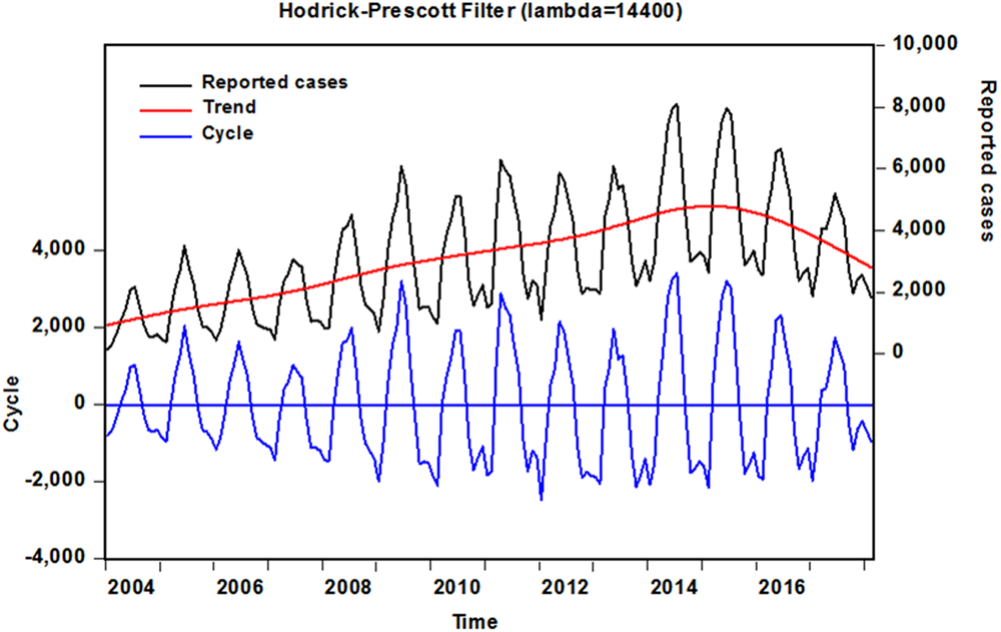
Decomposition of monthly human brucellosis time series in mainland China from 2004 to 2018 into trend and cyclical components using Hodrick-Prescott filter. Black line represents the reported cases. Red line stands for the decomposed long-term trend of the notified monthly cases of human brucellosis, suggesting that a slightly potential decline is observed between 2015 and February 2018. Blue line is the decomposed cyclical process with a circle of 12 months.

The data covered 170 observations from January 2004 to February 2018, a total of 4,491,081 reported cases with a monthly average incidence of 3,095 cases (average annual incidence rate was 0.0259 per 100,000 population), along with standard error of 144 cases over the whole period, 487,033 of whom occurred between 2004 and 2017, the morbidity cases elevated from 11,472 to 38,554 cases with an overall increase by 236.070% throughout the past decade. The incidence peak with 5,722 cases was witnessed in 2014, followed by 2015, the case numbers reached 56,989, which increased by 398.797% and 396.766% respectively than that of 2004 (Supplementary Fig. S1). When utilizing the Hodrick-Prescott decomposition technique to obtain the long-term trend and cyclical component of the observed incidence series from January 2004 to February 2018 (Fig. 1), it was found that notwithstanding a slightly potential decline existed between 2015 and February 2018, there still were a relatively higher reported cases compared with the earlier stages.

### Simulating and forecasting with the best-fitting ARIMA model

**Figure 2:**
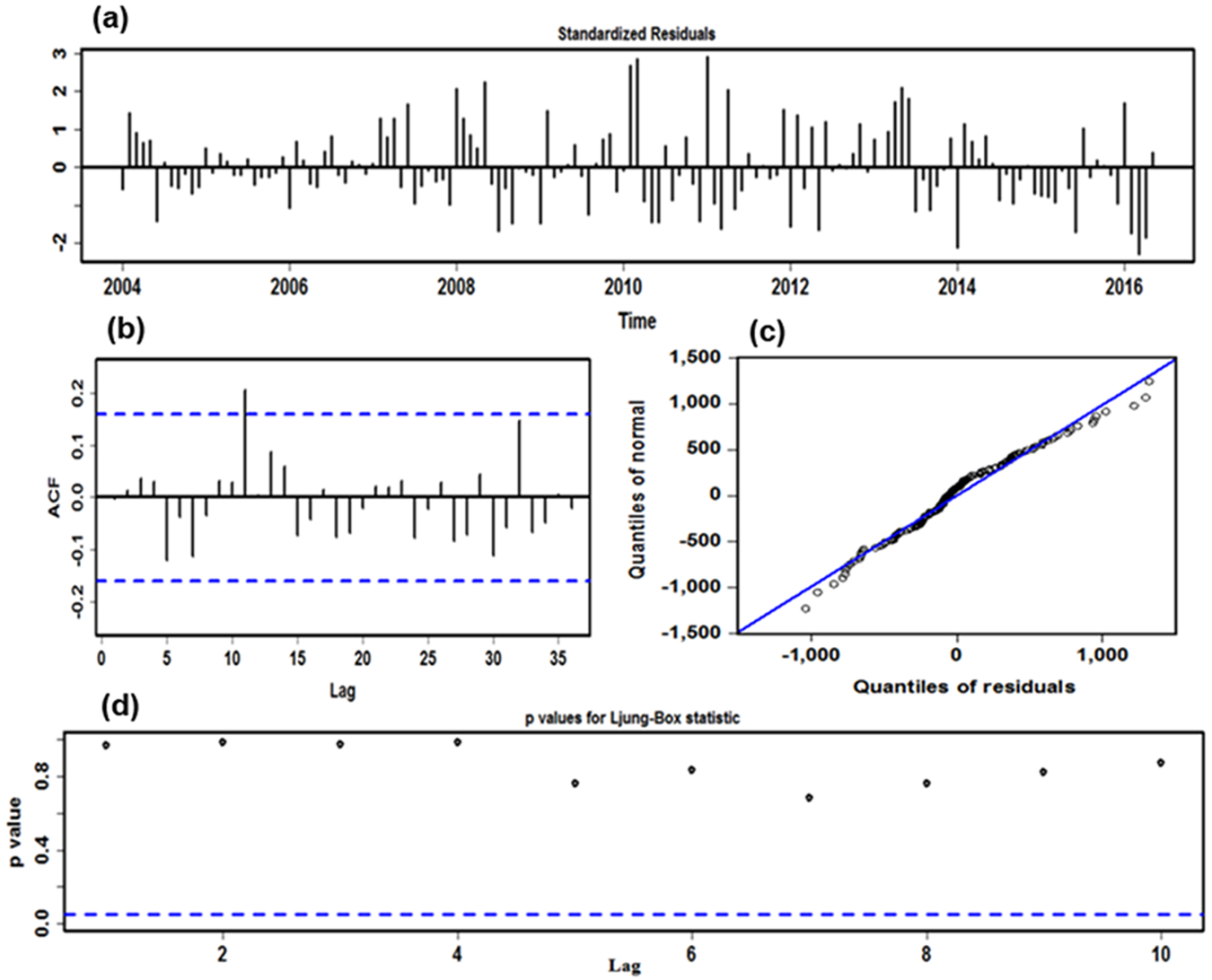
Residual diagnostic plots of ARIMA (1,1,1) × (0,1,1)_12_ model. (**a**) Standardized residuals. (**b**) Autocorrelation function (ACF) plot of the time-series residuals. (**c**) Normal Q-Q plot for determining the normality of the time-series residuals. (**d**) *P* values for Ljung-Box statistic. The ACF shows that no significant residual correlations are observed in the time series at the 5% level, and *P* values for Ljung-Box statistic are never significant at various lags. The normal Q-Q graph of the time-series residuals approximately falls along the line. These imply the selected ARIMA method offers a reasonable simulation to the monthly human brucellosis time series.

An ADF test (ADF = −1.734, *P* = 0.412) showed that monthly human brucellosis cases series between January 2004 and June 2017 was obviously non-stationary. Thus, a seasonal and non-seasonal difference was considered to remove the effects of seasonality and trends. After finalizing the level 1 differencing, a significant difference (ADF = −15.916, *P* < 0.001) was noted in the differenced sequence, the results revealed that the processing data were successfully stationary. Afterwards, in light of the spikes at different lags of the ACF and PACF graphs plotted with the seasonal adjustment sequence, several possible candidate models were roughly elected to further detect the best-fitting model by a trial-and-error approach (Supplementary Figs S2–S4 and Table S1). Finally, taking synthetically the correlations between the ACF and PACF graphs of the residual sequence, AIC, AICc, SBC as well as LL into consideration, the preferred model of ARIMA (1,1,1)(0,1,1)_12_ was yielded, where the error correlations at lags were approximately independent and normally distributed with zero means and variances, the residual series successfully attained white noise, while the ARCH effect was found at prior 18 lags in the residual series, the testing results of estimated parameters were all significant, and the values of the minimum AIC, AICc and SBC, as well as maximum LL were 2258.90, 2259.19, 2270.93, and −1125.46, respectively (Fig. 2 and Tables 1 and 2). The specified equation of the ARIMA model was written as (1 − B)(1 − B^12^)X_t_ = (1 + 0.869B) (1 + 0.617B^12^)ɛ_t/_(1 + 0.668B). Likewise, following the mentioned-above modeling steps, the human brucellosis cases series from January 2004 to December 2016 and June 2016 was respectively employed to verify the model uncertainty during the prediction: the best-simulating ARIMA model constructed using the first 156 data points was identified as an ARIMA (1,0,0)(0,1,1)_12_ specification, the parameter estimations and diagnostics for this model are revealed in Supplementary Figs S5–S8 and Tables S2–S4; and the best-fitting ARIMA method developed using the first 150 observations was still considered as an ARIMA (1,0,0)(0,1,1)_12_ specification, the parameter estimations and diagnostic checking for this model are presented in Supplementary Figs S9–S12 and Tables S5–S7. Next, these preferred models could be employed to perform their out-of-sample forecasting.

**Table 1:** Ljung-Box Q test of the residuals for each of the selected optimal models.

Lags	Residuals of ARIMA model		Residuals of ETS model		Residuals of hybrid model	
	Ljung-Box Q	*P*	Ljung-Box Q	*P*	Ljung-Box Q	*P*
1	0.002	0.969	0.000	0.990	0.807	0.369
3	0.225	0.973	0.339	0.953	3.671	0.299
6	2.800	0.833	13.479	0.036	7.022	0.319
9	5.141	0.822	18.900	0.026	9.690	0.376
12	12.180	0.431	33.050	0.001	11.413	0.494
15	14.892	0.459	37.469	0.001	12.601	0.633
18	16.199	0.579	42.457	0.001	15.396	0.635
21	17.127	0.703	44.570	0.002	19.761	0.536
24	18.414	0.782	47.079	0.003	20.694	0.657
27	19.932	0.834	49.803	0.005	24.873	0.582
30	23.542	0.792	53.888	0.005	30.349	0.448
33	29.190	0.657	58.505	0.004	32.664	0.484
36	29.713	0.761	58.715	0.010	35.564	0.489

**Table 2:** LM-test of the residuals for each of the selected optimal models.

Lags	Observed values		Residuals of ARIMA model		Residuals of ETS		Residuals of hybrid model	
	LM-test	*P*	LM-test	*P*	LM-test	*P*	LM-test	*P*
1	122.460	<0.001	15.779	<0.001	2.895	0.089	9.743	0.002
3	136.140	<0.001	16.211	0.001	4.494	0.213	11.230	0.011
6	134.050	<0.001	19.521	0.003	6.245	0.396	11.765	0.067
9	135.410	<0.001	19.347	0.022	0.396	0.570	11.789	0.226
12	136.460	<0.001	21.455	0.044	10.603	0.563	14.840	0.250
15	135.390	<0.001	31.128	0.008	13.110	0.594	22.274	0.101
18	132.880	<0.001	30.601	0.032	14.663	0.685	29.057	0.048
21	130.420	<0.001	31.324	0.068	17.190	0.700	32.522	0.052
24	127.500	<0.001	33.893	0.087	18.897	0.758	34.226	0.081
27	124.590	<0.001	35.623	0.124	20.894	0.791	35.623	0.124
30	121.780	<0.001	35.271	0.233	23.744	0.784	34.916	0.246
33	119.350	<0.001	37.754	0.261	26.049	0.800	35.490	0.352
36	117.290	<0.001	39.085	0.333	27.528	0.844	35.236	0.505

### Simulating and forecasting with the best-fitting ETS model

**Figure 3:**
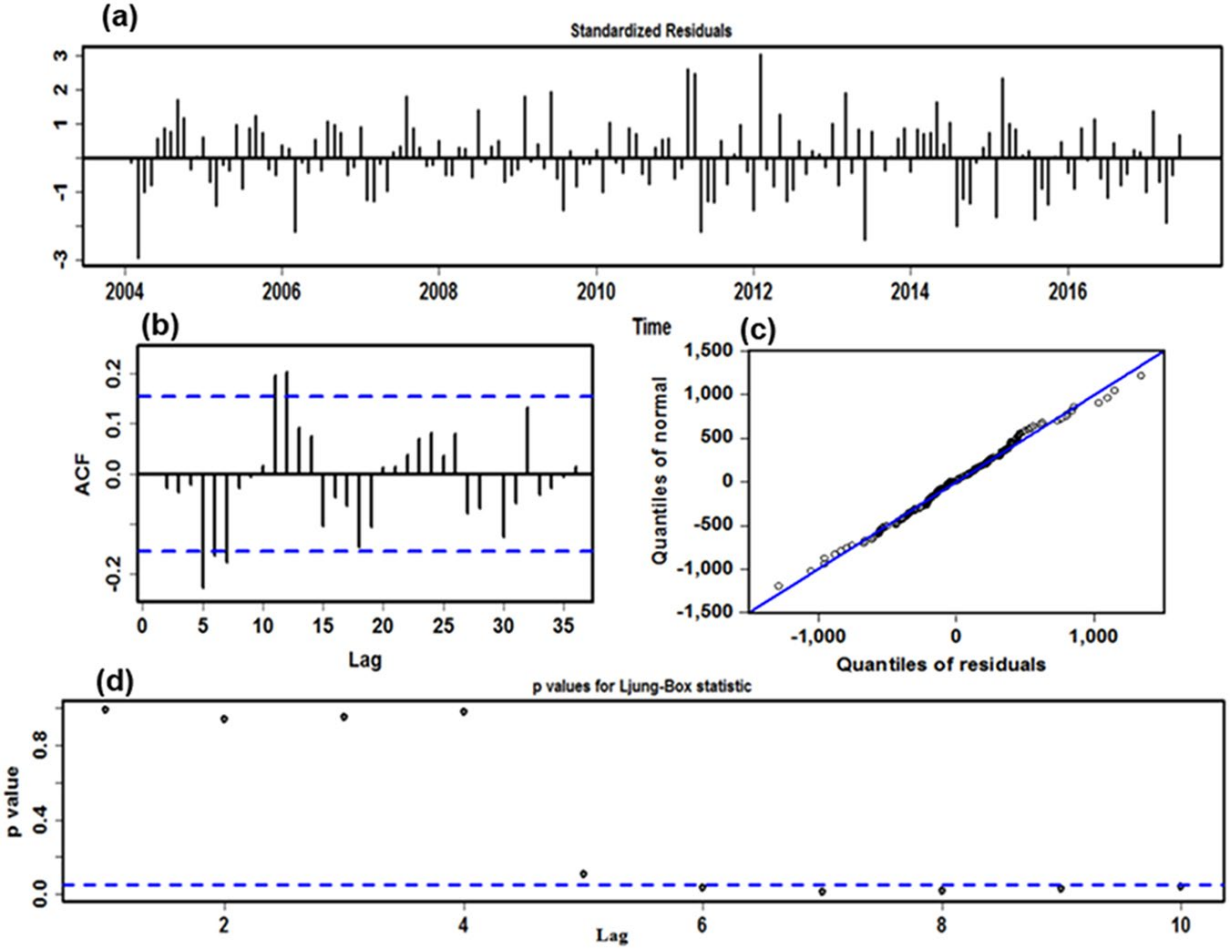
Residual diagnostic plots of ETS (A,N,A) model. (**a**) Standardized residuals. (**b**) Autocorrelation function (ACF) plot of the time-series residuals. (**c**) Normal Q-Q plot for determining the normality of the time-series residuals. (**d**) *P* values for Ljung-Box statistic. On the basis of the graphs, it seems that existing occult information are still required to explore.

As is shown in Supplementary Table S8, 30 potential candidate models were constructed to obtain the best-fitting ETS model, suggesting that the ETS (A,N,A) model with additive irregular fluctuation and additive seasonality was appropriate to accurately capture the included information of the monthly brucellosis series (Compact LL = −1397.564, Likelihood = −1215.337, AIC = 2823.129, BIC = 2866.355, HQ = 2840.679, AMSE = 385599.410), the estimated smoothing and initial parameters in sample simulating are shown in Supplementary Table S9. Diagnostic checking for the optimal ETS (A,N,A) model, it was found that the ACF graph of the residual sequence reserved individually dependent correlation at prior 12 lags, and the *P* values for Ljung-Box statistic were significantly difference after 2-stage lags, which documented existing occult information were still needed to exploit, yet the ARCH effect from the residuals was smoothed away by the preferred ETS (A,N,A) model (Fig. 3 and Tables 1 and 2). In parallel to the mentioned-above modeling procedures, in the two validation datasets, the best-mimicking ETS model established utilizing the data from January 2004 to December 2016 was viewed as an ETS (A,MD,M) form. As regards this selected model, all further statistical diagnostic results are displayed in Supplementary Fig. S13 and Tables S10 and S11. While the optimal ETS model erected with the data from January 2004 to June 2016 was thought of as an ETS (A,N,A) specification, and Supplementary Fig. S14 and Tables S12 and S13 provided an overview of the checking results for this preferred model. After choosing these best-fitting ETS models, they could be further used to calculate forecasts for individual out-of-sample.

### Simulating and forecasting with the best-fitting combined ARIMA-ETS model

**Figure 4:**
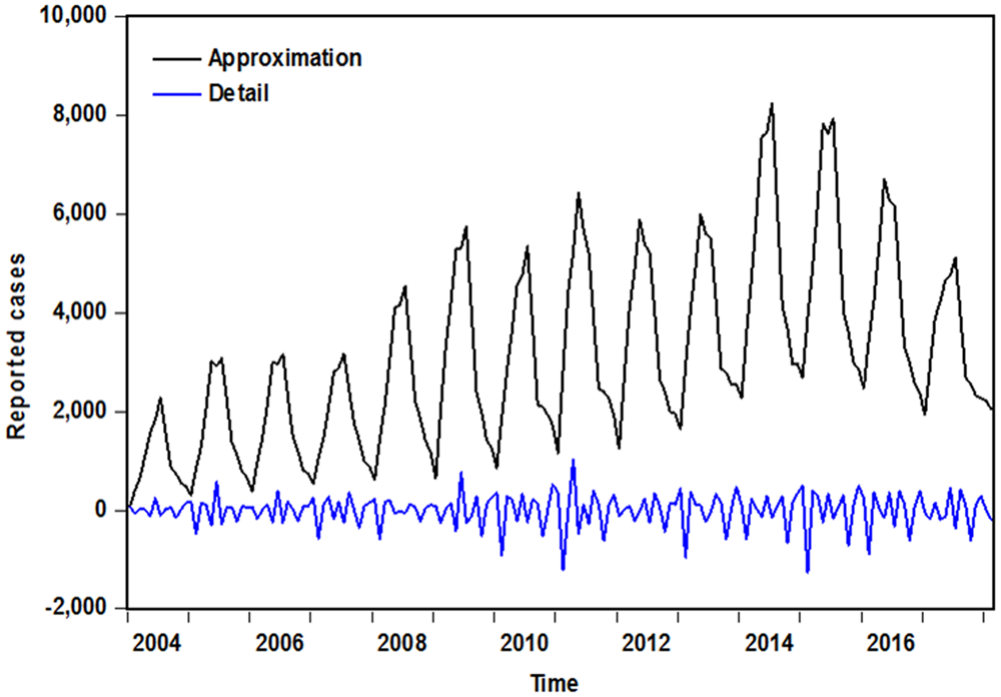
The approximations and details of human brucellosis morbidity decomposed by 1-level cof1 DWT. The plot demonstrates that the approximate component reserved the entire form of the human brucellosis morbidity cases time series, whereas the detailed component shows the remaining noise information.

**Figure 5:**
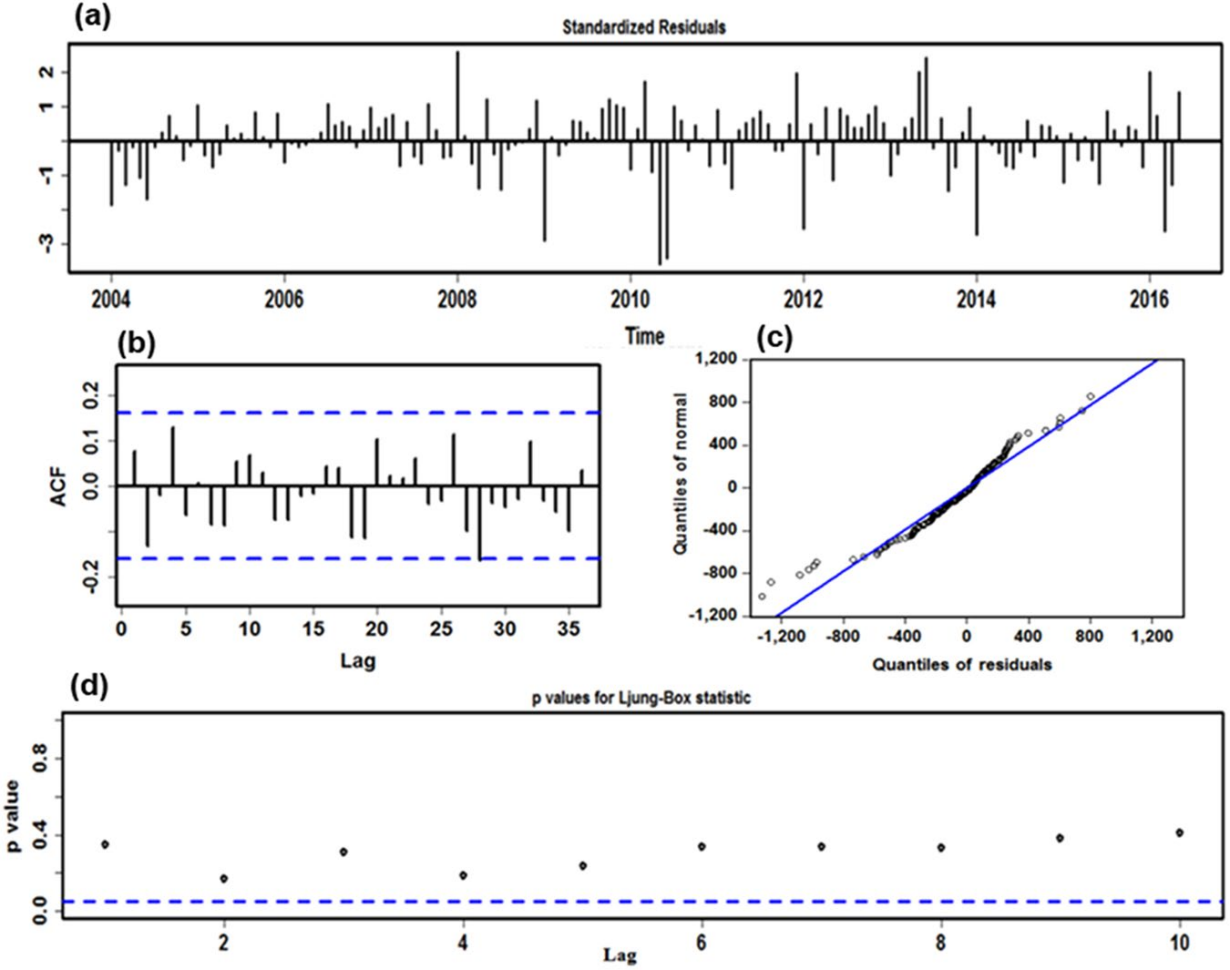
Residual diagnostic plots of hybrid ARIMA-ETS model. (**a**) Standardized residuals. (**b**) Autocorrelation function (ACF) plot of the time-series residuals. (**c**) Normal Q-Q plot for determining the normality of the time-series residuals. (**d**) *P* values for Ljung-Box statistic. Based on the plots, it can be found that the residuals seem to be well modeled as white noise, so this proposed hybrid approach is appropriate for fitting the data.

After the reported human brucellosis incidence series was split into approximation and detail using coif1 technique of one-dimensional DWT (Fig. 4), they were separately employed for building the ARIMA and ETS models, the procedure of model development was implemented as previously described. After undertaking, an ARIMA (0,1,2)(0,1,0)_12_ with a satisfactory diagnostic checking was identified as the preferred model for the decomposed approximation (Supplementary Table S14 and Fig. S15), and an ETS (A,N,A) model including additive error and additive seasonality was still regarded as the best-fitting model for the decomposed detail (Supplementary Tables S15 and S16). Then the simulations and forecasts of the hybrid ARIMA-ETS were comprised of the approximate and detailed parts fitted and predicted by the best-fitting basic ARIMA (0,1,2)(0,1,0)_12_ and ETS (A,N,A) models, respectively. The modeling performance diagnosis was still conducted in the in-sample fitted observations, the resulting data indicated all correlations fell within the confidence intervals and the *P* values of more than 0.05 for Ljung-Box statistic, exhibiting that the residuals are behaving like white noise and the included information of approximately normal distribution can be extracted based on the Q-Q plot of residuals (Fig. 5 and Table 1). In addition, the residual ARCH-effects existed in the observed series were largely ameliorated compared with those in the mimic data of the hybrid model (Table 2). Similarly, the datasets used to account for the model uncertainty were adopted to train the preferred hybrid techniques as mentioned before: the model combining an ARIMA (0,1,2) × (0,1,0)_12_ technique for the approximation-estimating and an ETS (A,N,A) specification for the detail-estimating constructed with the data from January 2004 to December 2016 should be elected as the optimal hybrid approach, and Supplementary Figs S16 and S17 and Tables S17–S19 displayed the summary statistics for the diagnostic checking of the best-fitting individual basic models. Whereas the optimal combined method built based on the observations from January 2004 to June 2016 was taken into consideration as such model that incorporated an ARIMA (0,1,2) × (1,1,0)_12_ approach for the approximation-forecasting and an ETS (A,N,A) specification for the detail-forecasting, and the results of the diagnostic analyses for this model can be seen in Supplementary Figs S18 and S19 and Tables S20–S22. Next, the selected combined models were further used to forecast the observations of their testing datasets.

### Comparison of simulating and forecasting accuracy

**Figure 6:**
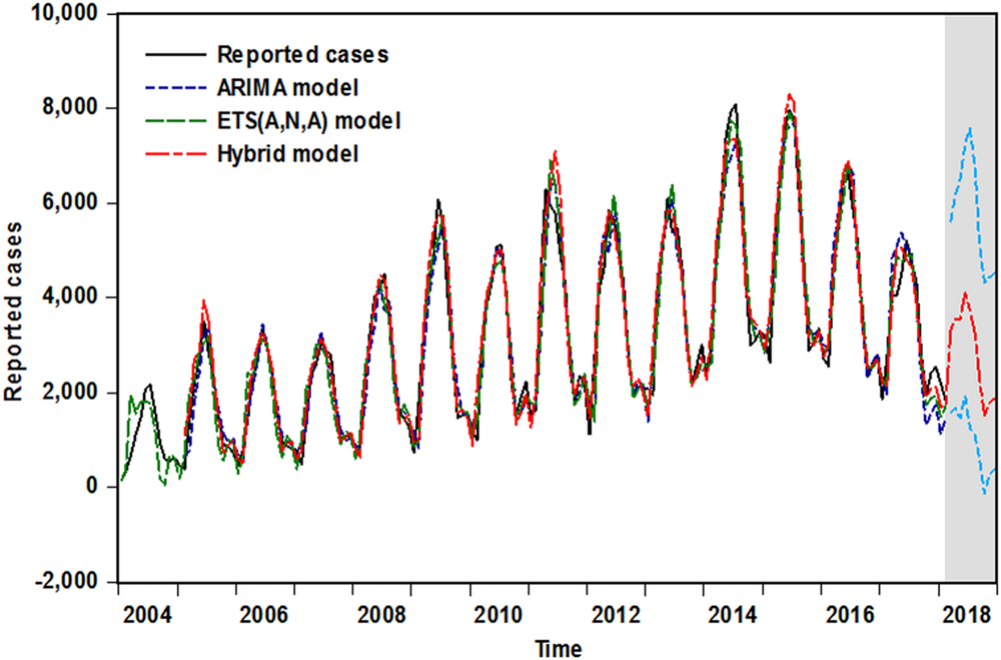
Comparison observations of in-sample fitting and out-of-sample forecast among selected models. The shaded area represents the incidence cases from March 2018 to December 2018 projected by the best-performing hybrid method, suggesting that a downward trend will continue to be observed in the near future.

Mutiple evaluating indicators are adopted to verify the in-sample fitting and out-of-sample forecasting performances among these selected optimal models. By comparison with the standard ARIMA and ETS techniques in the three forecasting intervals, it was found that the minimal values of the evaluation indicators involving in both training and testing sets were apparently observed in the hybrid ARIMA-ETS model (Table 3), and for the three established methods, taken as a whole, the mimic and predictive curves from the combined ARIMA-ETS technique were also in close proximity to the original data (Fig. 6), which further indicated that this combination model outperformed the basic ARIMA and ETS methods. Thus, the hybrid model was employed to attain the expected number of cases from March to December in 2018 (Table 4).

**Table 3:** Comparison results of in-sample fitting and out-of-sample forecasting performance for the selected models.

Models	Fitted efficacy				Forecasted efficacy			
	MAE	MAPE	RMSE	MER	MAE	MAPE	RMSE	MER
In-sample dataset from January 2004 to June 2017					8-step ahead forecasts			
ARIMA	346.116	0.136	454.333	0.105	598.325	0.248	658.392	0.210
ETS	345.315	0.167	438.405	0.111	361.498	0.143	421.175	0.127
Hybrid	253.541	0.117	353.722	0.077	237.417	0.088	304.676	0.083
Decreased percentages (%)								
ARIMA VS Hybrid	26.747	13.971	22.145	26.667	60.320	64.516	53.724	60.476
ETS VS Hybrid	26.577	29.940	19.316	30.631	34.324	38.462	27.660	34.646
In-sample dataset from January 2004 to December 2016					14-step ahead forecasts			
ARIMA	323.234	0.127	425.370	0.099	1430.380	0.451	1589.96	0.442
ETS	289.705	0.119	406.636	0.094	707.059	0.192	933.911	0.219
Hybrid	201.673	0.089	303.112	0.062	463.536	0.136	611.720	0.143
Decreased percentages (%)								
ARIMA VS Hybrid	37.608	29.921	28.742	37.374	67.593	69.845	61.526	67.647
ETS VS Hybrid	30.387	25.210	25.459	34.043	34.442	29.167	34.499	34.703
In-sample dataset from January 2004 to June 2016					20-step ahead forecasts			
ARIMA	321.300	0.130	423.246	0.099	1489.37	0.479	1662.42	0.441
ETS	312.309	0.131	414.286	0.102	985.483	0.329	1070.380	0.292
Hybrid	210.997	0.094	300.121	0.065	423.604	0.139	473.352	0.126
Decreased percentages (%)								
ARIMA VS Hybrid	34.330	27.692	29.091	35.000	71.558	70.981	71.526	71.429
ETS VS Hybrid	32.440	28.244	27.557	36.275	57.016	57.751	55.777	56.849

**Table 4:** Estimated values using combined model from March to December in 2018.

Date	Estimated values	95% confidence bounds
March	3301	[1595, 5615]
April	3558	[1682, 6160]
May	3553	[1463, 6516]
June	4108	[1928, 7253]
July	3737	[1261, 7589]
August	3244	[1091, 6758]
September	2273	[579, 5076]
October	1514	[−132, 4322]
November	1794	[276, 4441]
December	1875	[396, 4522]

## Discussion

Nowadays, brucellosis has still been deemed as a serious public-health problem owing to its resurgence in China and worldwide, we can not emphasize the importance of again initiating control strategies for this worsening status too much. While basic to any implementation of the prevention and elimination of this disease is the accurate forecasting for future epidemic trends. Thus, reported here is an extension of the basic ARIMA and ETS models to forecast the morbidity components included in infectious diseases, the constructed hybrid ARIMA-ETS approach based on the coif1 method of one-dimensional DWT was applied to grasp the temporal trends of human brucellosis incidence cases in mainland China. To date this is the only study to our best knowledge to explore the flexibility of combining the ARIMA and ETS models for predicting the brucellosis incidence in medical and health field. By analyzing different forecasting intervals, our results show that the predictive capacity and fitting efficiency of the combined ARIMA-ETS model can provide a notable improvement in the forecasting for the reported human brucellosis cases over the individual ARIMA and ETS approaches in the three forecasting horizons. The training residuals in the 8-step ahead forecasts for the MAE, MAPE, RMSE and MER indices decreased by 26.747%, 13.971%, 22.145% and 26.667% and the counterparts of testing residuals slumped by 60.320%, 64.516%, 53.724% and 60.476% respectively as compared with the corresponding parts of the basic ARIMA model. When used to compare with the basic ETS model, the reduced percentages of the training residuals for aforementioned four indices are 26.577%, 29.940%, 19.316% and 30.631% and the counterparts of testing residuals are 34.324%, 38.462%, 27.660% and 34.646%, respectively. In the same vein, in the 14-step and 20-step ahead forecasts, the values of these indices are rather lower than that of the single ARIMA and ETS methods. And as illustrated in Fig. 6, the fitting and prediction values of combined ARIMA-ETS model also revealed a fairly similar ascending and descending trends to the actual human brucellosis incidence. These findings suggest that the hybrid ARIMA-ETS model can not only better track the internal rules and epidemic characteristics of the original observations but also retain a robust stability in the medium and long-term predictions. It is clear that this combined technique built can be a helpful tool for further understanding the future temporal distribution of human brucellosis incidence. However, of note, with the considerable development of combination model, currently, numerous hybrid techniques have already been applied to function as an advanced warning for communicable diseases, such as combining the ARIMA model with a radical basis function model^31^, back-propagation neural network^32^, generalized regression neural network^33^, nonlinear autoregressive neural network^23^ and autoregressive conditional heteroscedasticity^34^, all of which meet the expectations for individual forecasts. Thus, in order to preferably facilitate some targeted control and eradication programmes for human brucellosis in mainland China, the model used in this study should be compared with aforementioned methods to identify the best-performing model-fitting. Besides, in our study, in the 8-step and 20-step ahead forecasts, we found that the modeling scale-dependent measures (RMSE and MAE) in the ARIMA model were slightly inferior to those in the ETS model, while the modeling measures derived from the percentage errors (MAPE and MER) were mildly superior to the counterparts in the ETS model, the finding is not in the line of earlier literature^20^ which concluded that the ETS method provided a higher estimation accuracy than the ARIMA approach in the morbidity prediction of pertussis. By contrast, the forecasting corresponding four performance indices in the ETS model were remarkably lower than that in the ARIMA model. With respect to this discrepancy, a contributory factor is that several point simulations of the ETS model are largely far away from the actual. Furthermore, also suggesting that it is necessary to explore suitable prediction methods for different data.

As we all know, seasonal identification will be a key step towards implementing the prevention strategies for brucellosis^22^. The results from our study implied that an evident seasonality was found in the months of March, April, May, June, July and August during covering 14 years, accounting for 75.025% of total incidence, among which the reported cases in June leave much to be desired, accounting for 19.659% of subjects occurred in high-risk seasonality, similar seasonal characteristics are also presented in other countries^2,35^. Moreover, the outbreaks ordinarily exist during the 6 months as well^2,35,36^. So far, no study has indicated that brucellosis can be transmitted among humans. Accordingly, the drastically increasing susceptible sheep and goats especially in grassland areas, frequent circulation of unpasteurized and unquarantined affected livestock products from brucellosis-endemic areas to non-endemic, variation of pathogenic strains, changing climatic factors, and the prosperities of tourism in these months may mainly be responsible for the high-risk seasonality in China^3,4,37^. Since the mid-1990s, with the re-emergence of brucellosis, which has captured national attention, quite a few measures have then been taken to curb and harness the occurrence of brucellosis on the Chinese mainland, and a slightly downward trend was observed until 2015. However, whether a short-term rebound in morbidity will occur, as previous study reported^3^, remains still unknown. Thus we construct a hybrid model with the best-fitting and -predicting performance for the aggregated data spanning 14 years in China to mimic the epidemic trends in the near future. Admittedly, a exhilarating finding was that the morbidity of human brucellosis seems to emerge a obvious plunge in the subsequent 10 months of 2018, and as compared to an earlier study^3^, our approach gets a more clear perspective of epidemic trends of human brucellosis. Nevertheless, the expected cases of human brucellosis are still relatively high and present, manifesting China is afflicted with a chronic threat of brucellosis.

Some demerits, even though the established combined model achieves satisfactory mimic and predictive capabilities, should be pointed out in our present study. Firstly, the aggregated morbidity cases utilized in this work were obtained from national passive infectious disease surveillance, which makes it difficult to well control the quality of data due to potentially existing under-reporting, misdiagnosis and delay^6^, the actual morbidity cases of human brucellosis might thus be much more than the monitored. Nevertheless, the reported data made a real reflection on brucellosis to the foremost extent^38^, indicating that our comprehensive forecasts are still considered to mirror the present real epidemic trends of human brucellosis morbidity on the Chinese mainland. Secondly, the 1-level coif1 wavelet was only applied to decompose the original observations. Thirdly, although the hybrid model developed is applicable for medium- and long-term predictions in a morbidity series, in practice, the up-to-data incidence cases should also be continuously collected to verify the extrapolation performance of the hybrid model, in order to make updates in time. Lastly, the hybrid model was established based on the countrywide data in the period 2004–2017. Therefore, the findings obtained merely stand for the overall epidemic trends of human brucellosis in mainland China. Re-modeling for location-specific incidence data might serve as guidance to the implementation of specific public health planning, and whether the model is suitable for forecasting other kinds of infectious diseases remains to be re-validated.

Taken together, on the one hand, we have established a new hybrid model that can efficiently identify and extract the features of human brucellosis incidence contained and overcome the limitations from single model, it may be a rewarding tool to add a new sphere to our understanding of the future epidemic trends of human brucellosis in mainland China, and assist medical decision maker in rationally allocating health resources and appropriately developing the preventive and control measures for human brucellosis in mainland China. On the other hand, although a forecasted downward trend may be observed in the following months of 2018, the morbidity cases are still comparatively high and present, enhancing the awareness of ongoing prevention and control for this disease is not only necessary, but also indispensible.

## Materials and Methods

### Data collection

The monthly incidence cases of human brucellosis time series from January 1, 2004 to February 31, 2018 were collated and summarized from the Chinese Center for Disease Control and Prevention (CDC) (http://www.nhfpc.gov.cn/jkj/s3578/new_list.shtml), and the website of Disease Surveillance (http://www.jbjc.org/CN/ article/showVolumnList.do). The ethical approval or consent fails to be warranted for our present study as the monthly surveillance data of human brucellosis are publicly available in China.

### Establishing ARIMA model

To date the ARIMA model has always been deemed as a classical time series method for forecasting the morbidity of infectious diseases^39,40^. When an ARIMA model was utilized to fit time series data, the processing steps provide a helpful general procedure. (1) Identification of model. The prerequisite using an ARIMA model is that the time series must be a stationary series with a mean of zero. Thus, an Augmented Dickey-Fuller (ADF) test^19^ is firstly implemented to detect whether the series possesses unit root or not, and for a non-stationary series, the effects of season and trend are supposed to be removed to obtain ameliorated data by Box-Cox transformation or differencing^41^. (2) Estimation and diagnosis of model. The best-fitting model should be searched for with the suitable criteria of the minimal schwarz bayesian criterion (SBC), akaike information criterion (AIC), corrected akaike information criterion (AICc) or maximum log-likelihood (LL) function^20^. Once an optimal model has been sought out, the residual parts should be testified as white noise with autocorrelation and partial autocorrelation functions falling approximately within the 95% confidence intervals around zero and the estimated parameters being statistically significant. (3) Calculating forecasts. After finalizing the construction of preferred model, then 1-step- to multi-step-ahead predictions can be calculated recursively. An ARIMA (p, d, q) (P, D, Q)_s_ model can be expressed as^33^

1 $$\,\{\begin{array}{l}{\rm{\varphi }}({\rm{B}}){\rm{\Phi }}({{\rm{B}}}^{{\rm{s}}}){\nabla }^{{\rm{d}}}{\nabla }_{s}^{D}\,{X}_{t}={\rm{\theta }}({\rm{B}}){\rm{\Theta }}({{\rm{B}}}^{{\rm{s}}}){{\rm{\varepsilon }}}_{{\rm{t}}}\\ {\rm{E}}({{\rm{\varepsilon }}}_{{\rm{t}}})=0,\,{\rm{Var}}({{\rm{\varepsilon }}}_{{\rm{t}}})={\sigma }_{\varepsilon }^{2},\,{\rm{E}}({{\rm{\varepsilon }}}_{{\rm{t}}}\,{{\rm{\varepsilon }}}_{{\rm{s}}})=0,\,{\rm{s}}\ne {\rm{t}}\\ {\rm{E}}({{\rm{x}}}_{{\rm{s}}}{{\rm{\varepsilon }}}_{{\rm{t}}})=0,\,{\forall }_{s} < {\rm{t}}\end{array}$$

Here, B is the backward shift operator, ɛ_t_ is the residuals from time series, S stands for the periodicity of the original data, d and D denote the non-seasonal and seasonal differenced times, respectively. p and q denote the order of autoregressive model and moving average model, respectively. P and Q denote the order of seasonal autoregressive model and moving average model, respectively. ${\nabla }^{{\rm{d}}}$ = (1 − B)^d^, ${\nabla }_{s}^{D}$ = (1 − B^s^)^D^, ϕ(B) = 1 − ϕ_1_B-…-ϕ_p_B^p^, θ(B) = 1 − θ_1_B-…-θ_q_B^q^, Ф(B^s^) = 1 − Ф_1_B^s^-…- Ф_P_B^Ps^, Θ(B^s^) = 1 − Θ_1_B^s^-…-Θ_Q_B^Qs^.

### Establishing ETS model

The Error-Trend-Seasonal (ETS) model nested the classical ES model into a dynamic nonlinear model framework using state-space based likelihood calculations with 30 potential choices on the basis of decomposed components of trend, seasonality, and error for infectious diseases, which extraordinarily contributes to forecasting a canonical time series with different components^29,42–44^. The included underlying features of an ETS model can be specified as the following pattern^29^

2 $$\,\{\begin{array}{ll}{\rm{E}} & \{{\rm{A}},\,{\rm{M}}\}\\ {\rm{T}} & \{{\rm{N}},\,{\rm{A}},\,{\rm{M}},\,{\rm{AD}},\,{\rm{MD}}\}\\ {\rm{S}} & \{{\rm{N}},\,{\rm{A}},\,{\rm{M}}\}\end{array}$$

Here, E = error, T = trend, S = seasonality, N = none, A = additive, M = multiplicative, AD = additive dampened, and MD = multiplicative dampened (dampened term utilizes an added parameter to abate the influence of the secular trend over time), which can shape a total of candidate 30 ETS models associated with aforementioned varying choices. For obtaining the optimal model from 30 possible models, Likelihood based comparisons can be carried out employing the standard likelihood based criteria: AIC, BIC, average mean square error (AMSE), Hannan-Quinn Criterion (HQ), or the LL function^29^. Ultimately, among the AIC, BIC, HQ, and AMSE minimizing, coupled with the LL function maximizing the indices across all available models is the best-mimic model adopted.

### Establishing combined ARIMA and ETS model based on coif1 wavelet

To well capture what behind the morbidity time series of brucellosis, motivated by the merits of single model^25,28^, a hybrid ARIMA-ETS model based coif1 wavelet was proposed to effectively forecast the future secular changes of brucellosis incidence series. In the first step, the coif1 approach of one-dimensional DWT was applicable for decomposing the observed brucellosis series into the approximation representing the high-scale, low-frequency information of the observations and detail symbolizing the low-scale, high-frequency information of the observations^25,45,46^. Next, the approximate subset was simulated and predicted by an ARIMA method; the detailed subset was fitted and forecasted by an optimal ETS model. Finally, the mimic and forecasting results of the combined ARIMA-ETS model were written as

3 $${\mathop{X}\limits^{\frown {}}}_{i}={a}_{i}+{d}_{i}$$

where ${\mathop{X}\limits^{\frown {}}}_{i}$ refers to the mimic and forecasted incidence with combined model, *a*_*i*_ denote the modeling and predictions of approximations with ETS model, *d*_*i*_ is the stimulations and forecasts of detailed subset with ARIMA model.

### Assessing model performance

In order to distinguish the stimulation and forecasting accuracy from the selected various models, the root mean square error (RMSE), mean absolute error (MAE), mean error rate (MER), and mean absolute percentage error (MAPE) were primarily applied to measure the performance accuracy among the three selected optimal models.

4 $${\rm{RMSE}}=\sqrt{\frac{1}{N}\sum _{i=1}^{N}{({X}_{i}-{\mathop{X}\limits^{\frown {}}}_{i})}^{{\rm{2}}}}$$

5 $${\rm{MAE}}=\frac{1}{N}\sum _{i=1}^{N}|{X}_{i}-{\mathop{X}\limits^{\frown {}}}_{i}|$$

6 $${\rm{MER}}=\frac{\frac{1}{N}\sum _{i=1}^{N}|{X}_{i}-{\mathop{X}\limits^{\frown {}}}_{i}|}{{\bar{X}}_{i}}$$

7 $${\rm{MAPE}}=\frac{1}{N}\sum _{i=1}^{N}\frac{|{X}_{i}-{\mathop{X}\limits^{\frown {}}}_{i}|}{{X}_{i}}$$

Here, *X*_*i*_ represents the actual reported cases, ${\mathop{X}\limits^{\frown {}}}_{i}$ refers to the mimic and forecasted incidence with selected models, ${\bar{X}}_{i}$ denotes the average of actual reported cases, *N* stands for the number of mimics and forecasts.

### Statistic process

During the development of models process, in order to validate the model uncertainty in multi-step ahead forecasts, three forecasting horizons were considered in the present work. Therefore, the reported observed values (170 data points) of human brucellosis from January 2004 to February 2018 were classified into three parts, among which the first 162 (from January 2004 to June 2017), 156 (from January 2004 to December 2016), and 150 observations (from January 2004 to June 2017) were specified as the training datasets, respectively; whereas the remaining 8 (from July 2017 to February 2018), 14 (from January 2017 to February 2018) and 20 observations (from July 2016 to February 2018) were assigned as the testing datasets, respectively. The Lagrangian multiplier (LM) and Ljung-Box Q tests were employed to verify the conditional heteroskedastic behaviour and volatility (ARCH effect) and stochasticity (white noise) from the residuals of in-sample modeling for the selected optimal models, respectively, All statistical analyses were mainly implemented with Eviews10.0 software (IHS, Inc. USA) and R statistical package (version 3.4.3, R Development Core Team, Vienna, Austria). With cut-off for statistical significance set at a two-sided *P* value < 0.05.

## Electronic supplementary material

Supplementary information

## Data Availability

They are available, please contact the correspondence author or the first author to obtain the available data.
