# Supplementary material for: Temporal trends analysis of human brucellosis incidence in mainland China from 2004 to 2018
Source: Sci Rep. 2018 Oct 26;8:15901. doi: 10.1038/s41598-018-33165-9 (PMC6203822; doi:10.1038/s41598-018-33165-9)
Supplement: Supplementary file 1 — Supplementary information [file 41598_2018_33165_MOESM1_ESM.pdf]

Supplementary information: Temporal trends analysis of human brucellosis incidence in mainland China from 2004 to 2018

Yongbin Wang[1,+], Chunjie Xu[2,+], Shengkui Zhang[1], Zhende Wang[1], Ying Zhu[1], Juxiang Yuan[1,]*

[1] School of Public Health, North China University of Science and Technology, Tangshan, Hebei Province, P.R. China; 568019636@qq.com(Y.B.W.); 1527317477@qq.com(S.K.Z.); 9002629472@qq.com (Z.D.W.); 121545724@qq.com(Y.Z.)

[2] School of Public Health, Capital Medical University, Beijing 100069, P.R. China; 2248209559@qq.com(C.J.X.)

* Corresponding author: Juxiang Yuan, No.21 Bohai Road, Caofeidian Xincheng, Tangshan City, Hebei Province, 063210, P.R. China; Tel: +86-315-8805002; e-mail: yuanjx@ncst.edu.cn

[+] These authors contributed equally to this work

Supplementary Figure and Table captions

Figure S1. Time series of monthly human brucellosis incidence cases in mainland China from 2004 to 2018.

Figure S2. Temporal characteristics of monthly human brucellosis incidence cases series in mainland China from January 2004 to June 2017 with the first-order non-seasonal difference (d=1). (**a**) The time series after the first-order non-seasonal difference. (**b**) Autocorrelation function(ACF) plot of the differenced series. (**c**) Partial autocorrelation function(PACF) plot of the differenced series. Based on the graphs, showing that this differenced series smoothes away the changing trends in the original time series, yet there still exists evident seasonal pattern in the differenced series.

Figure S3. Temporal characteristics of monthly human brucellosis incidence cases series in mainland China from January 2004 to June 2017 with the first-order seasonal difference(D=1). (**a**) The time series after the first-order seasonal difference. (**b**) Autocorrelation function(ACF) plot of the differenced series. (**c**) Partial autocorrelation function(PACF) plot of the differenced series. Based on the graphs, we might well consider the differenced series to be a stationary series.

Figure S4. Temporal characteristics of monthly human brucellosis incidence cases series in mainland China from January 2004 to June 2017 with the first-order non-seasonal(d=1) and seasonal differences(D=1). (**a**) The time series after the first-order non-seasonal and seasonal differences. (**b**) Autocorrelation function(ACF) plot of the differenced series. (**c**) Partial autocorrelation function(PACF) plot of the differenced series. The plots exhibite that the residuals are independently distributed aside from the correlation at lag 11, this also is reasonable as two higher-order functions may occasionally exceed the estimated confidence intervals.

Figure S5. Temporal characteristics of monthly human brucellosis incidence cases series in mainland China from January 2004 to December 2016 with the first-order non-seasonal difference (d=1). (**a**) The time series after the first-order non-seasonal difference. (**b**) Autocorrelation function(ACF) plot of the differenced series. (**c**) Partial autocorrelation function(PACF) plot of the differenced series. The graphs manifest that the differenced series looks much more stationary when compared with the original time series, however, there still is evident seasonal pattern in the differenced series.

Figure S6. Temporal characteristics of monthly human brucellosis incidence cases series in mainland China from January 2004 to December 2016 with the first-order seasonal difference(D=1). (**a**) The time series after the first-order seasonal difference. (**b**) Autocorrelation function(ACF) plot of the differenced series. (**c**) Partial autocorrelation function(PACF) plot of the differenced series. On the basis of the graphs, we might well view the differenced series as a stationary series.

Figure S7. Temporal characteristics of monthly human brucellosis incidence cases series in mainland China from January 2004 to December 2016 with the first-order non-seasonal(d=1) and seasonal difference(D=1). (**a**) The time series after the first-order non-seasonal and seasonal differences. (**b**) Autocorrelation function(ACF) plot of the differenced series. (**c**) Partial autocorrelation function(PACF) plot of the differenced series. The plots demonstrate that the residuals are independently distributed aside from the correlation at lag 11, this also is reasonable as two higher-order functions may occasionally exceed the estimated confidence bounds.

Figure S8. Residual diagnostic plots of the ARIMA(1,0,0)$(0,1,1)_{12}$ model for monthly human brucellosis incidence cases series in mainland China from January 2004 to December 2016. (**a**) Standardized residuals. (**b**) Autocorrelation function(ACF) plot of the time-series residuals. (**c**)

Normal Q-Q plot for determining the normality of the time-series residuals. (**d**) *P* values for Ljung-Box statistic. The residuals are almost normally and independently distributed and *P* values for Ljung-Box statistic are more than 0.05, indicating that the selected model is fairly suitable.

Figure S9. Temporal characteristics of monthly human brucellosis incidence cases series in mainland China from January 2004 to June 2016 with the first-order non-seasonal difference (d=1). (**a**) The time series after the first-order non-seasonal difference. (**b**) The autocorrelation function(ACF) plot of the differenced series. (**c**) The partial autocorrelation function(PACF) plot of the differenced series. Based on the graphs, showing that this differenced series removes the changing trends in the original time series, yet there still exists evident seasonal pattern in the differenced series.

Figure S10. Temporal characteristics of monthly human brucellosis incidence cases series in mainland China from January 2004 to June 2016 with the first-order seasonal difference(D=1). (**a**) The time series after the first-order seasonal difference. (**b**) The autocorrelation function(ACF) plot of the differenced series. (**c**) The partial autocorrelation function(PACF) plot of the differenced series. Based on the graphs, we might well consider the differenced series to be a stationary series.

Figure S11. Temporal characteristics of monthly human brucellosis incidence cases series in mainland China from January 2004 to June 2016 with the first-order non-seasonal(d=1) and seasonal difference(D=1). (**a**) The time series after the first-order non-seasonal and seasonal differences. (**b**) Autocorrelation function(ACF) plot of the differenced series. (**c**) Partial autocorrelation function(PACF) plot of the differenced series. The plots exhibit that the residuals are independently distributed aside from the correlation at lag 11, this also is reasonable as two higher-order functions may occasionally exceed the estimated confidence intervals.

Figure S12. Residual diagnostic plots of the ARIMA(1,0,0)×(0,1,1) $_{12}$ model for monthly human brucellosis incidence cases series in mainland China from January 2004 to June 2016. (**a**) Standardized residuals. (**b**) Autocorrelation function(ACF) plot of the time-series residuals. (**c**) Normal Q-Q plot for determining the normality of the time-series residuals. (**d**) *P* values for Ljung-Box statistic. As can be seen from the plots above, the ACF of the residuals shows no apparent departure from the model assumptions and the Q-statistic is never significant at various lags, indicating that this selected ARIMA model seems to fit well for the time series.

Figure S13. Residual diagnostic plots of the ETS(A,MD,M) model for monthly human brucellosis incidence cases series in mainland China from January 2004 to December 2016. (**a**) Standardized residuals. (**b**) Autocorrelation function(ACF) plot of the time-series residuals. (**c**) Normal Q-Q plot for determining the normality of the time-series residuals. (**d**) *P* values for Ljung-Box statistic. From the graphs above we can see that the residuals are close to be normal, the autocorrelation can be observed at lags 1, 11, and 32, and the *P* values are large except for lags 1 and 2, suggesting that the elected ETS model seemingly can be enhanced.

Figure S14. Residual diagnostic plots of the ETS(A,N,A) model for monthly human brucellosis incidence cases series in mainland China from January 2004 to June 2016. (**a**) Standardized residuals. (**b**) Autocorrelation function(ACF) plot of the time-series residuals. (**c**) Normal Q-Q plot for determining the normality of the time-series residuals. (**d**) *P* values for Ljung-Box statistic. As shown in figures above, which indicate that it may be possible to improve the prediction.

Figure S15. Residual diagnostic plots of the ARIMA(0,1,2)×(0,1,0)$_{12}$ model for approximation generated by coif1 technique from January 2004 to June 2017. (**a**) Standardized residuals. (**b**) Autocorrelation function(ACF) plot of the time-series residuals. (**c**) Partial autocorrelation

function(PACF) plot of the time-series residuals. (**d**) *P* values for Ljung-Box statistic. All correlations fall within the estimated 95% uncertainty limits and a large *P* value for Ljung-Box statistic is observed demonstrating that the model is appropriate.

Figure S16. Residual diagnostic plots of the ARIMA$(0,1,2)\times(0,1,0)_{12}$ model for approximation generated by coif1 technique from January 2004 to December 2016. (**a**) Standardized residuals. (**b**) Autocorrelation function(ACF) plot of the time-series residuals. (**c**) Normal Q-Q plot for determining the normality of the time-series residuals. (**d**) *P* values for Ljung-Box statistic. There is not significant correlation in the prediction errors and the results for Ljung-Box statistic look good (more than 0.05), indicating that this ARIMA model seems to simulate the time series well.

Figure S17. Residual diagnostic plots of the hybrid ARIMA-ETS model for monthly human brucellosis incidence cases series in mainland China from January 2004 to December 2016. (**a**) Standardized residuals. (**b**) Autocorrelation function(ACF) plot of the time-series residuals. (**c**) Normal Q-Q plot for determining the normality of the time-series residuals. (**d**) *P* values for Ljung-Box statistic. The error correlations at lags were approximately independent and the *P* values for Ljung-Box statistic are more than 0.05 apart from the values at lags 1 and 2. It seems that this hybrid method can be employed to fit the data.

Figure S18. Residual diagnostic plots of the ARIMA$(0,1,2)\times(1,1,0)_{12}$ model for approximation generated by coif1 technique from January 2004 to June 2016. (**a**) Standardized residuals. (**b**) Autocorrelation function(ACF) plot of the time-series residuals. (**c**) Normal Q-Q plot for determining the normality of the time-series residuals. (**d**) *P* values for Ljung-Box statistic. The ACF of the residuals shows no apparent departure from the model assumptions, and the Q-statistic is never significant at the lags, suggesting that this is a suitable model for the time series.

Figure S19. Residual diagnostic plots of the hybrid ARIMA-ETS model for monthly human brucellosis incidence cases series in mainland China from January 2004 to June 2016. (**a**) Standardized residuals. (**b**) Autocorrelation function(ACF) plot of the time-series residuals. (**c**) Normal Q-Q plot for determining the normality of the time-series residuals. (**d**) *P* values for Ljung-Box statistic. It can be seen from the plots above that this hybrid model appears to fit well except for the fact that a distribution with heavy tails than the normal distribution should be employed.

Table S1. The estimated parameters and performance indexes of selected candidate models based on original observations from January 2004 to June 2017.

Table S2. The estimated parameters and performance indexes of the selected candidate models based on the original observations from January 2004 to December 2016.

Table S3. Ljung-Box Q test of the time-series residuals from January 2004 to December 2016 for each of the selected optimal models.

Table S4. ARCH LM-test of the time-series residuals from January 2004 to December 2016 for each of the selected optimal models.

Table S5. The estimated parameters and performance indexes of the selected candidate models based on the original observations from January 2004 to June 2016.

Table S6. Ljung-Box Q test of the time-series residuals from January 2004 to June 2016 for each of the selected optimal models.

Table S7. ARCH LM-test of the time-series residuals from January 2004 to June 2016 for each of the selected optimal models.

Table S8. Comparison results of in-sample goodness of fit for the candidate ETS specifications.

Table S9. Initial parameters of ETS(A,N,A) model.

Table S10. Comparison results of in-sample goodness of fit from January 2004 to December 2016 for the candidate ETS specifications.

Table S11. Initial parameters of ETS(A,MD,M) model.

Table S12. Comparison results of in-sample goodness of fit from January 2004 to June 2016 for the candidate ETS specifications.

Table S13. Initial parameters of ETS(A,N,A) model.

Table S14. The estimated parameters and performance indexes of selected candidate models based on approximation from January 2004 to June 2017.

Table S15. Comparison results of in-sample goodness of fit for the candidate ETS specifications.

Table S16. Initial parameters of ETS(A,N,A) model.

Table S17. The estimated parameters and performance indexes of selected candidate models based on approximation from January 2004 to December 2016.

Table S18. Comparison results of goodness of fit for the candidate ETS specifications based on detail from January 2004 to December 2016.

Table S19. Initial parameters of ETS(A,N,A) model.

Table S20. The estimated parameters and performance indexes of selected candidate models based on approximation from January 2004 to June 2016.

Table S21. Comparison results of goodness of fit for the candidate ETS specifications based on detail from January 2004 to June 2016.

Table S22. Initial parameters of ETS(A,N,A) model.

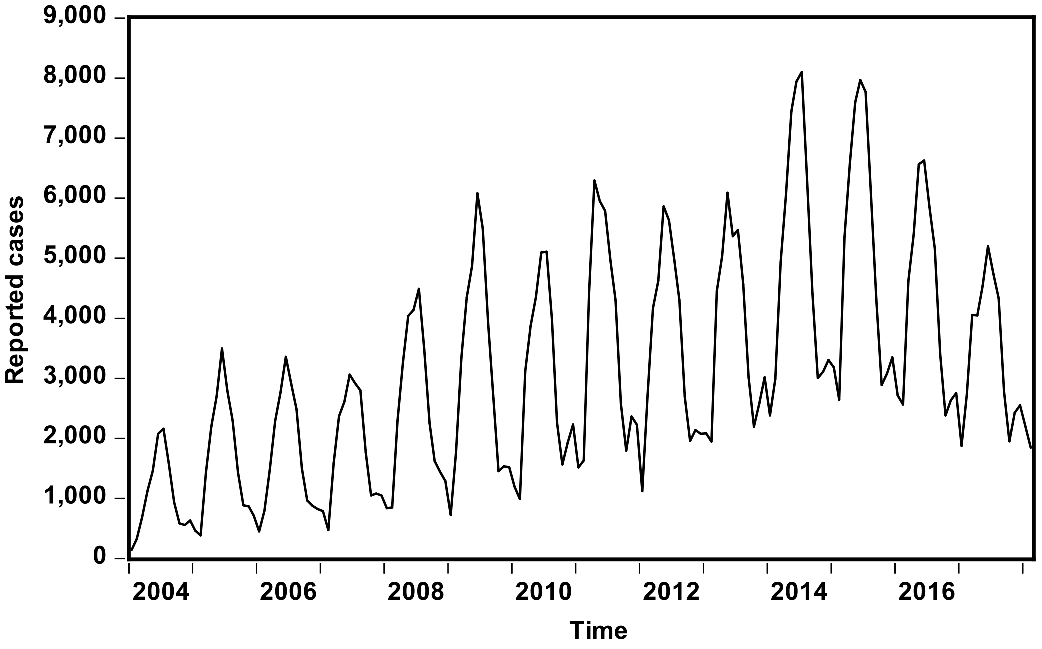

Figure S1. Time series of monthly human brucellosis incidence cases in mainland China from 2004 to 2018.

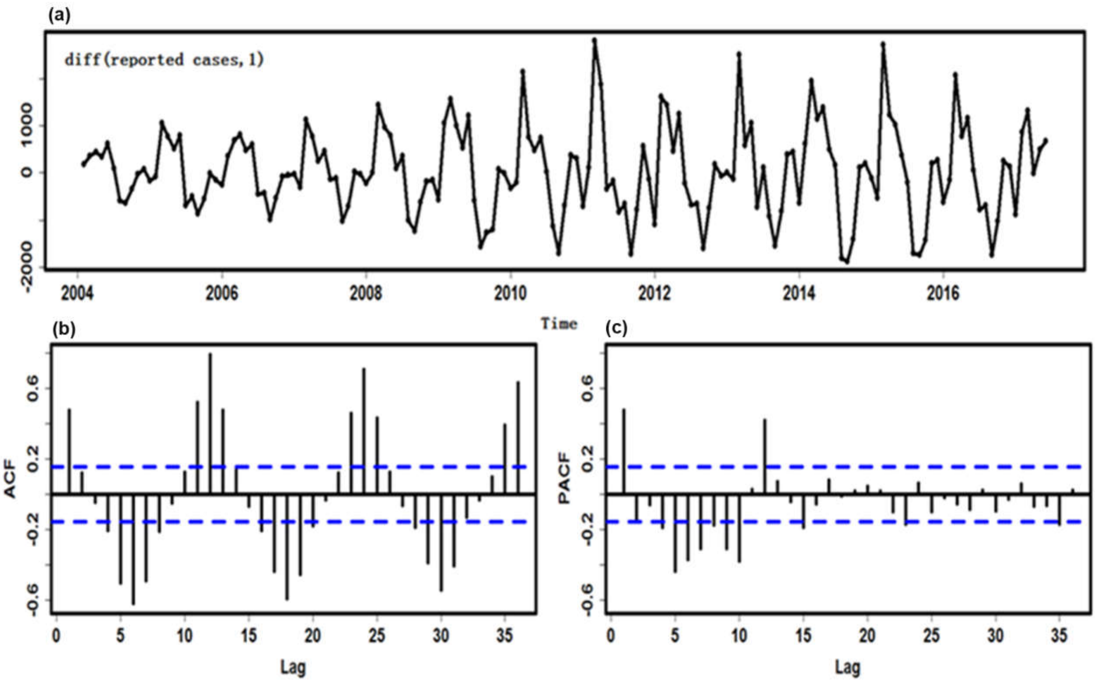

Figure S2. Temporal characteristics of monthly human brucellosis incidence cases series in mainland China from January 2004 to June 2017 with the first-order non-seasonal difference (d=1). (**a**) The time series after the first-order non-seasonal difference. (**b**) Autocorrelation function(ACF) plot of the differenced series. (**c**) Partial autocorrelation function(PACF) plot of the differenced series. Based on the graphs, showing that this differenced series smoothes away the changing trends in the original time series, yet there still exists evident seasonal pattern in the differenced series.

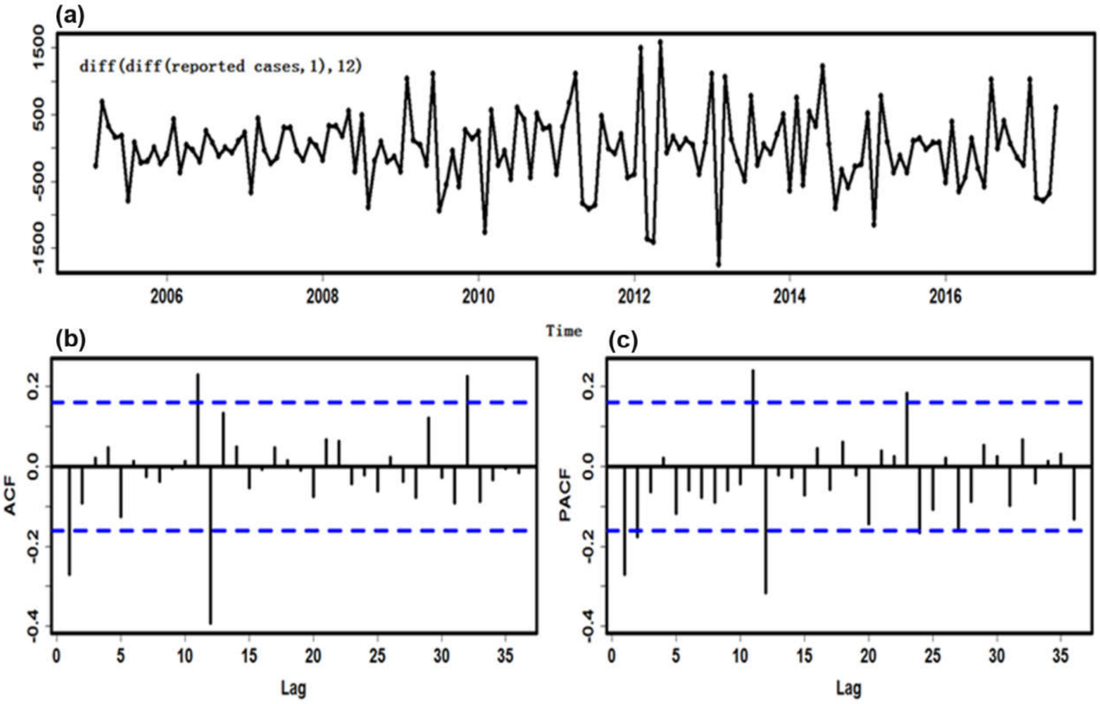

Figure S3. Temporal characteristics of monthly human brucellosis incidence cases series in mainland China from January 2004 to June 2017 with the first-order seasonal difference(D=1). (**a**) The time series after the first-order seasonal difference. (**b**) Autocorrelation function(ACF) plot of the differenced series. (**c**) Partial autocorrelation function(PACF) plot of the differenced series. Based on the graphs, we might well consider the differenced series to be a stationary series.

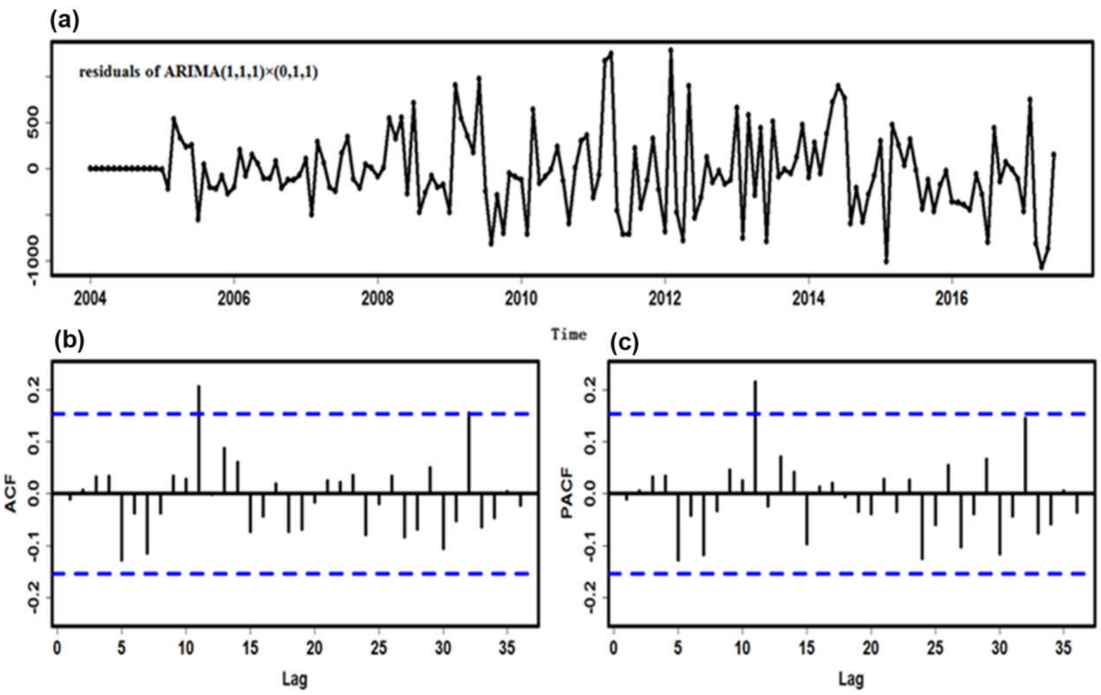

Figure S4. Temporal characteristics of monthly human brucellosis incidence cases series in mainland China from January 2004 to June 2017 with the first-order non-seasonal(d=1) and seasonal differences(D=1). (**a**) The time series after the first-order non-seasonal and seasonal differences. (**b**) Autocorrelation function(ACF) plot of the differenced series. (**c**) Partial autocorrelation function(PACF) plot of the differenced series. The plots exhibite that the residuals are independently distributed aside from the correlation at lag 11, this also is reasonable as two higher-order functions may occasionally exceed the estimated confidence intervals.

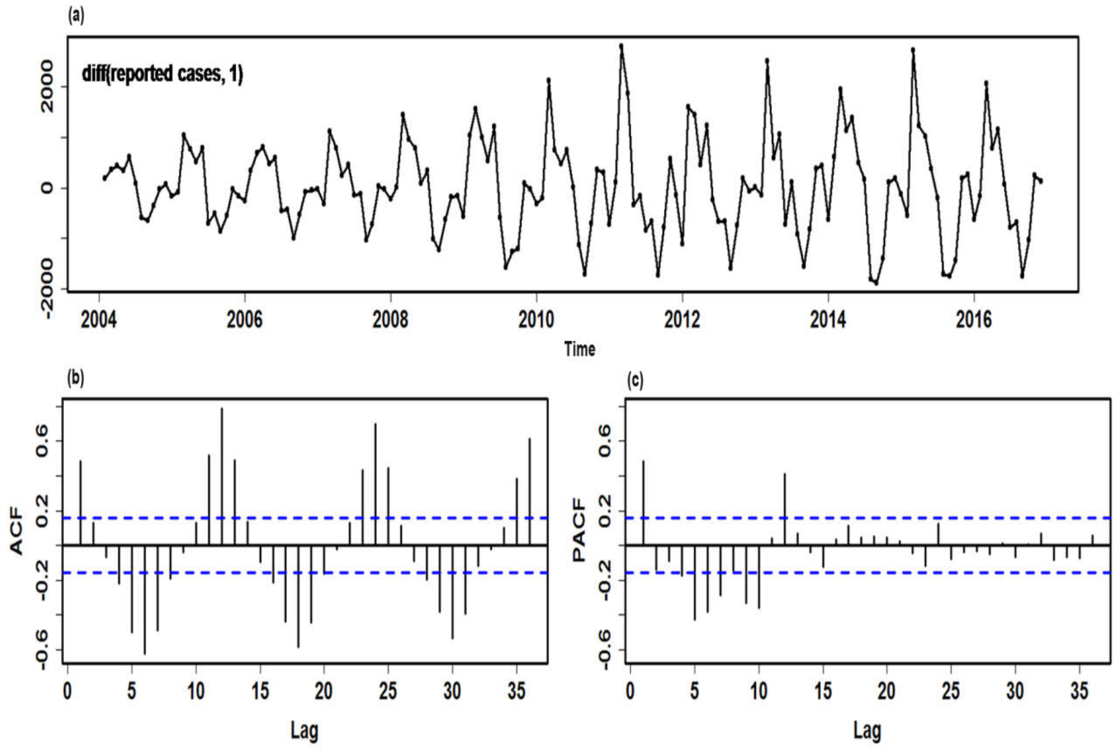

Figure S5. Temporal characteristics of monthly human brucellosis incidence cases series in mainland China from January 2004 to December 2016 with the first-order non-seasonal difference (d=1). (**a**) The time series after the first-order non-seasonal difference. (**b**) Autocorrelation function(ACF) plot of the differenced series. (**c**) Partial autocorrelation function(PACF) plot of the differenced series. The graphs manifest that the differenced series looks much more stationary when compared with the original time series, however, there still is evident seasonal pattern in the differenced series.

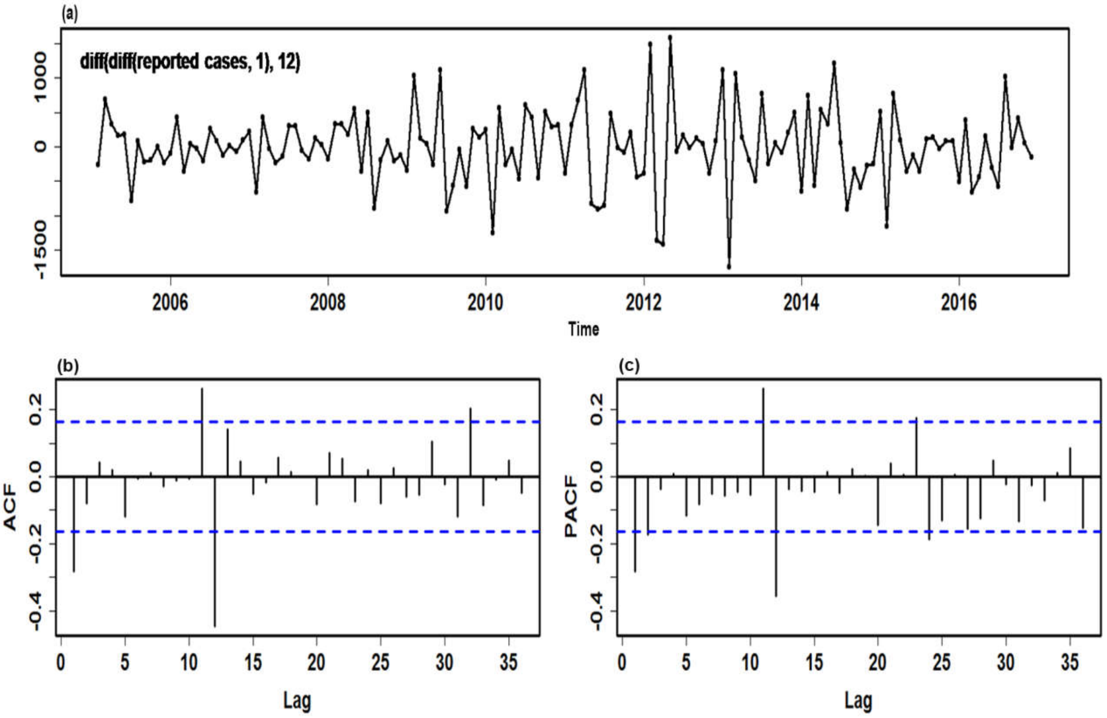

Figure S6. Temporal characteristics of monthly human brucellosis incidence cases series in mainland China from January 2004 to December 2016 with the first-order seasonal difference(D=1). (**a**) The time series after the first-order seasonal difference. (**b**) Autocorrelation function(ACF) plot of the differenced series. (**c**) Partial autocorrelation function(PACF) plot of the differenced series. On the basis of the graphs, we might well view the differenced series as a stationary series.

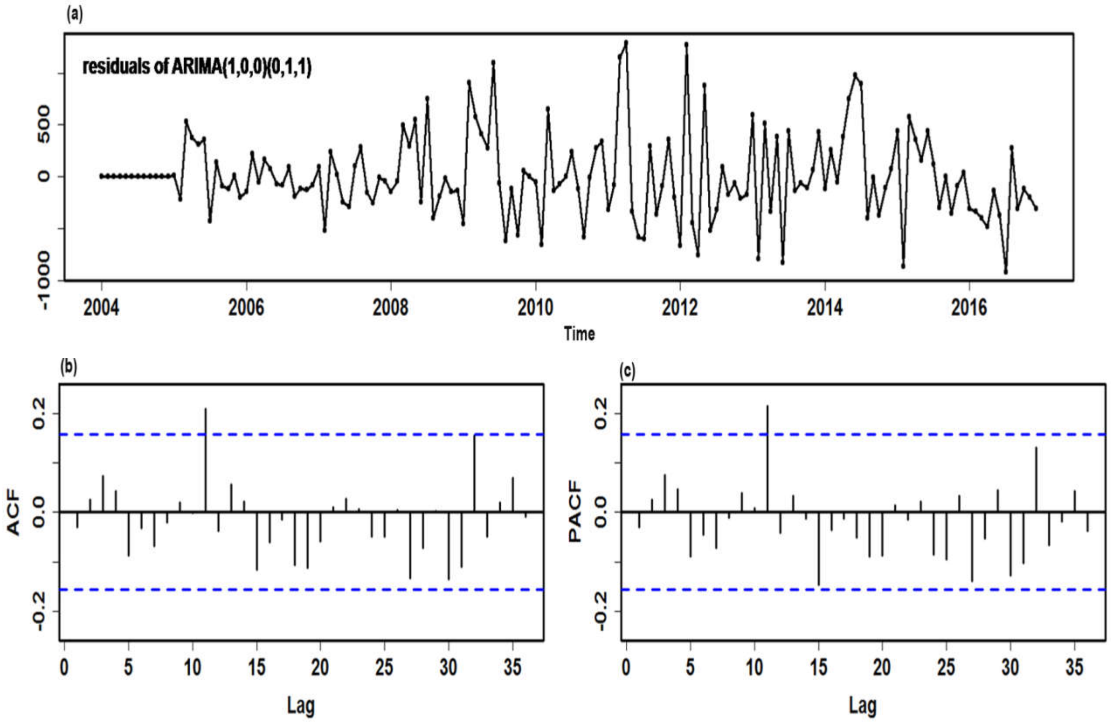

Figure S7. Temporal characteristics of monthly human brucellosis incidence cases series in mainland China from January 2004 to December 2016 with the first-order non-seasonal(d=1) and seasonal difference(D=1). (**a**) The time series after the first-order non-seasonal and seasonal differences. (**b**) Autocorrelation function(ACF) plot of the differenced series. (**c**) Partial autocorrelation function(PACF) plot of the differenced series. The plots demonstrate that the residuals are independently distributed aside from the correlation at lag 11, this also is reasonable as two higher-order functions may occasionally exceed the estimated confidence bounds.

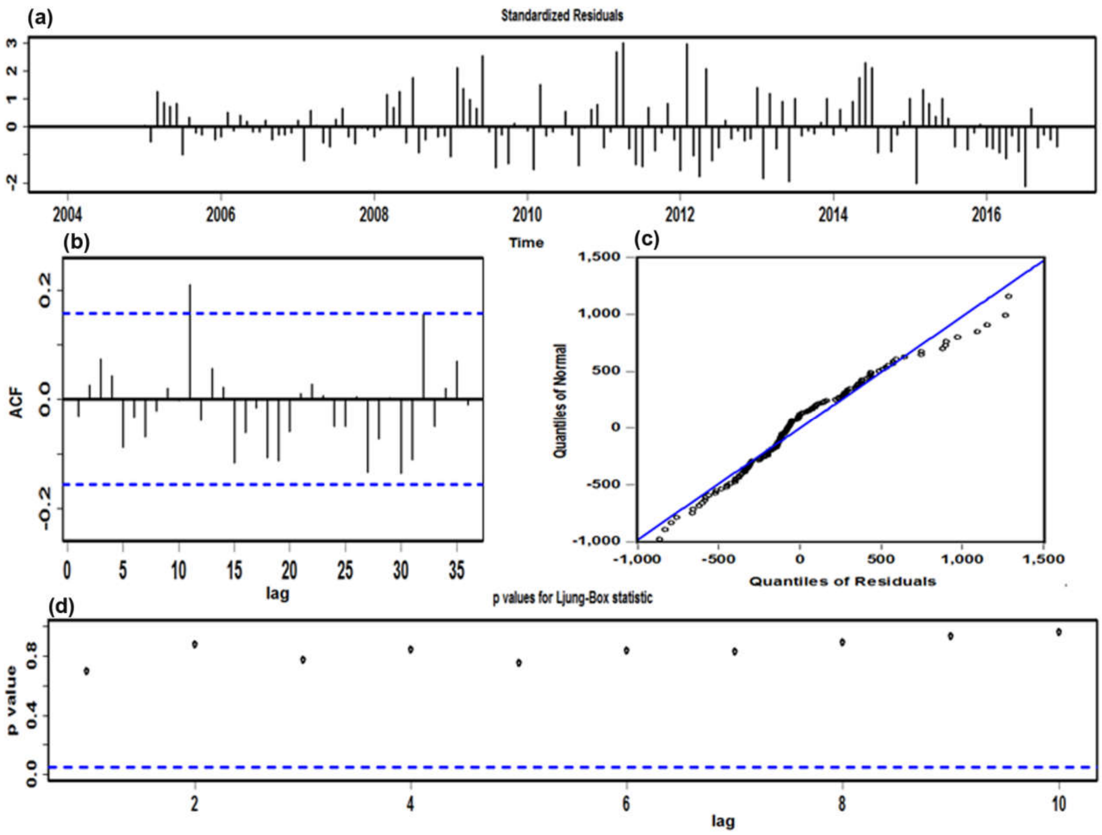

Figure S8. Residual diagnostic plots of the ARIMA(1,0,0)(0,1,1)$_{12}$ model for monthly human brucellosis incidence cases series in mainland China from January 2004 to December 2016. (**a**) Standardized residuals. (**b**) Autocorrelation function(ACF) plot of the time-series residuals. (**c**) Normal Q-Q plot for determining the normality of the time-series residuals. (**d**) *P* values for Ljung-Box statistic. The residuals are almost normally and independently distributed and *P* values for Ljung-Box statistic are more than 0.05, indicating that the selected model is fairly suitable.

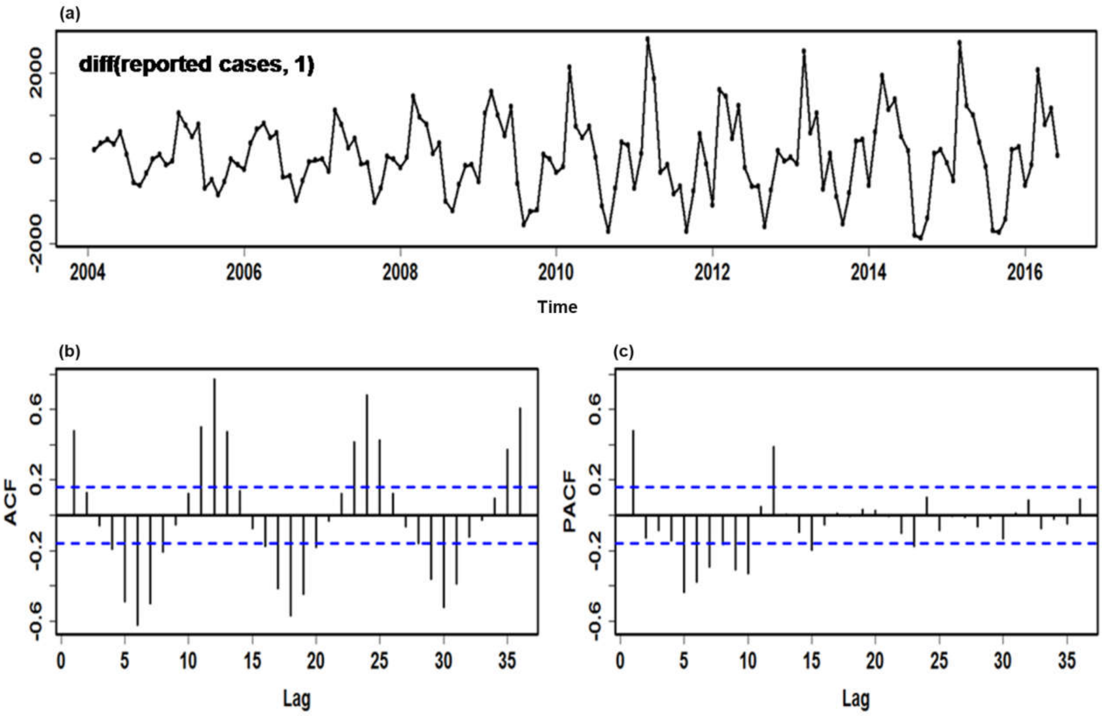

Figure S9. Temporal characteristics of monthly human brucellosis incidence cases series in mainland China from January 2004 to June 2016 with the first-order non-seasonal difference (d=1). (**a**) The time series after the first-order non-seasonal difference. (**b**) The autocorrelation function(ACF) plot of the differenced series. (**c**) The partial autocorrelation function(PACF) plot of the differenced series. Based on the graphs, showing that this differenced series removes the changing trends in the original time series, yet there still exists evident seasonal pattern in the differenced series.

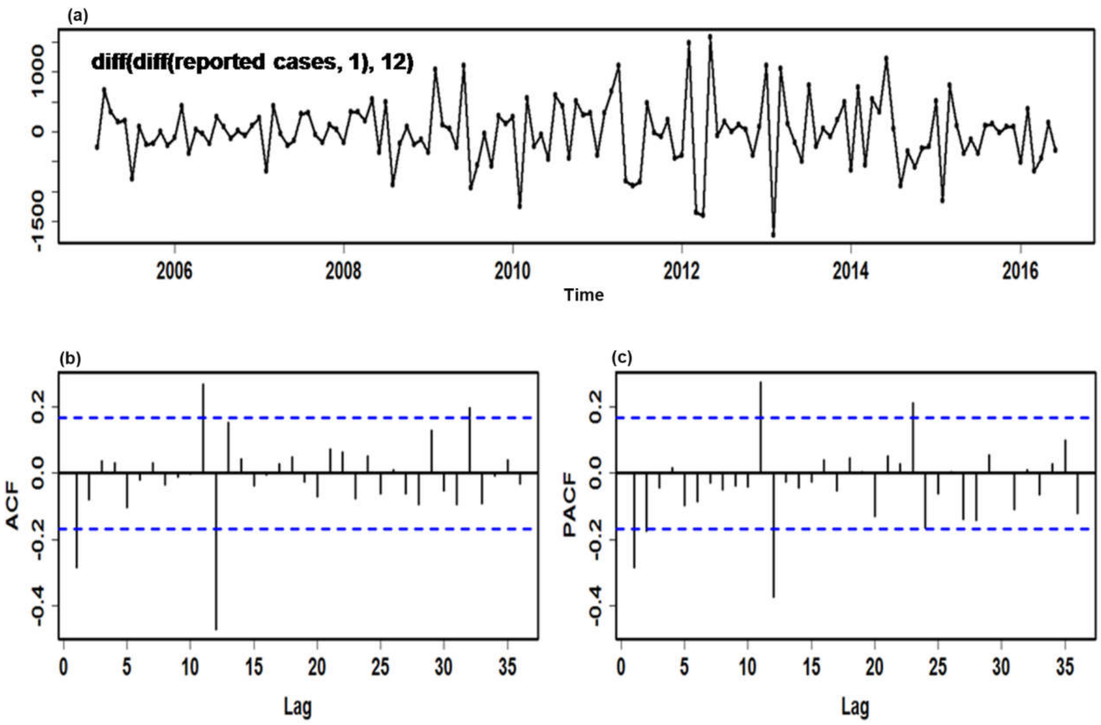

Figure S10. Temporal characteristics of monthly human brucellosis incidence cases series in mainland China from January 2004 to June 2016 with the first-order seasonal difference(D=1). (**a**) The time series after the first-order seasonal difference. (**b**) The autocorrelation function(ACF) plot of the differenced series. (**c**) The partial autocorrelation function(PACF) plot of the differenced series. Based on the graphs, we might well consider the differenced series to be a stationary series.

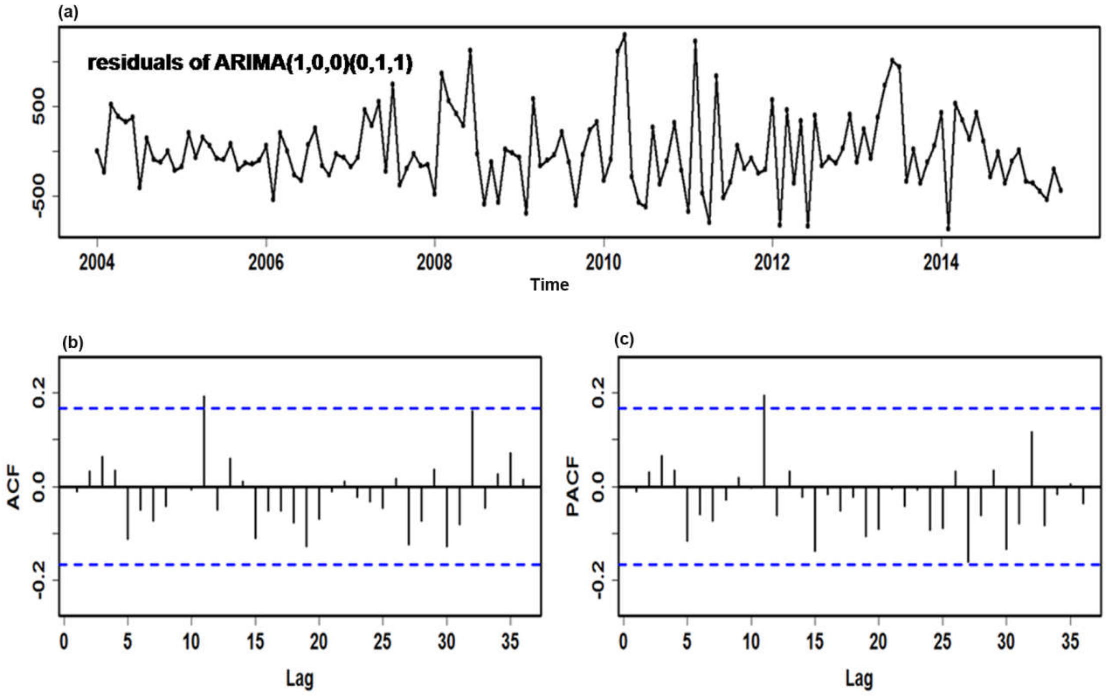

Figure S11. Temporal characteristics of monthly human brucellosis incidence cases series in mainland China from January 2004 to June 2016 with the first-order non-seasonal(d=1) and seasonal difference(D=1). (**a**) The time series after the first-order non-seasonal and seasonal differences. (**b**) Autocorrelation function(ACF) plot of the differenced series. (**c**) Partial autocorrelation function(PACF) plot of the differenced series. The plots exhibit that the residuals are independently distributed aside from the correlation at lag 11, this also is reasonable as two higher-order functions may occasionally exceed the estimated confidence intervals.

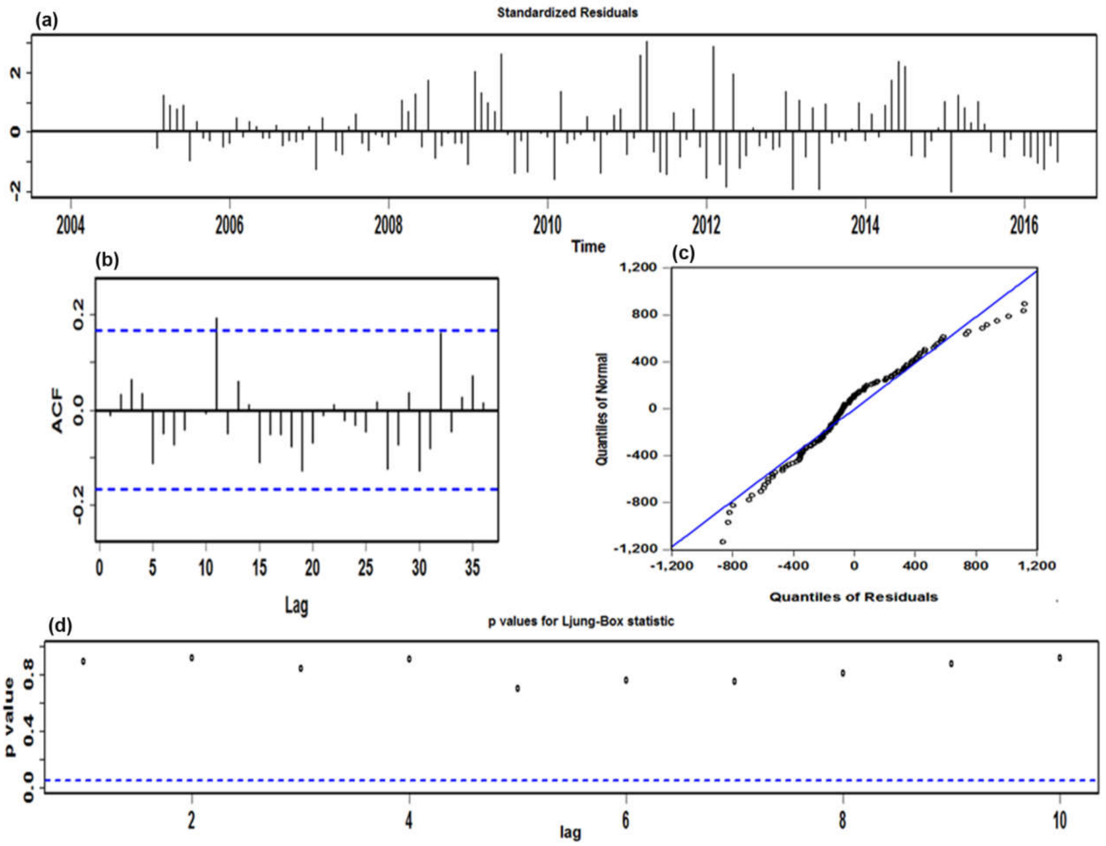

Figure S12. Residual diagnostic plots of the ARIMA(1,0,0)×(0,1,1) $_{12}$ model for monthly human brucellosis incidence cases series in mainland China from January 2004 to June 2016. (**a**) Standardized residuals. (**b**) Autocorrelation function(ACF) plot of the time-series residuals. (**c**) Normal Q-Q plot for determining the normality of the time-series residuals. (**d**) *P* values for Ljung-Box statistic. As can be seen from the plots above, the ACF of the residuals shows no apparent departure from the model assumptions and the Q-statistic is never significant at various lags, indicating that this selected ARIMA model seems to fit well for the time series.

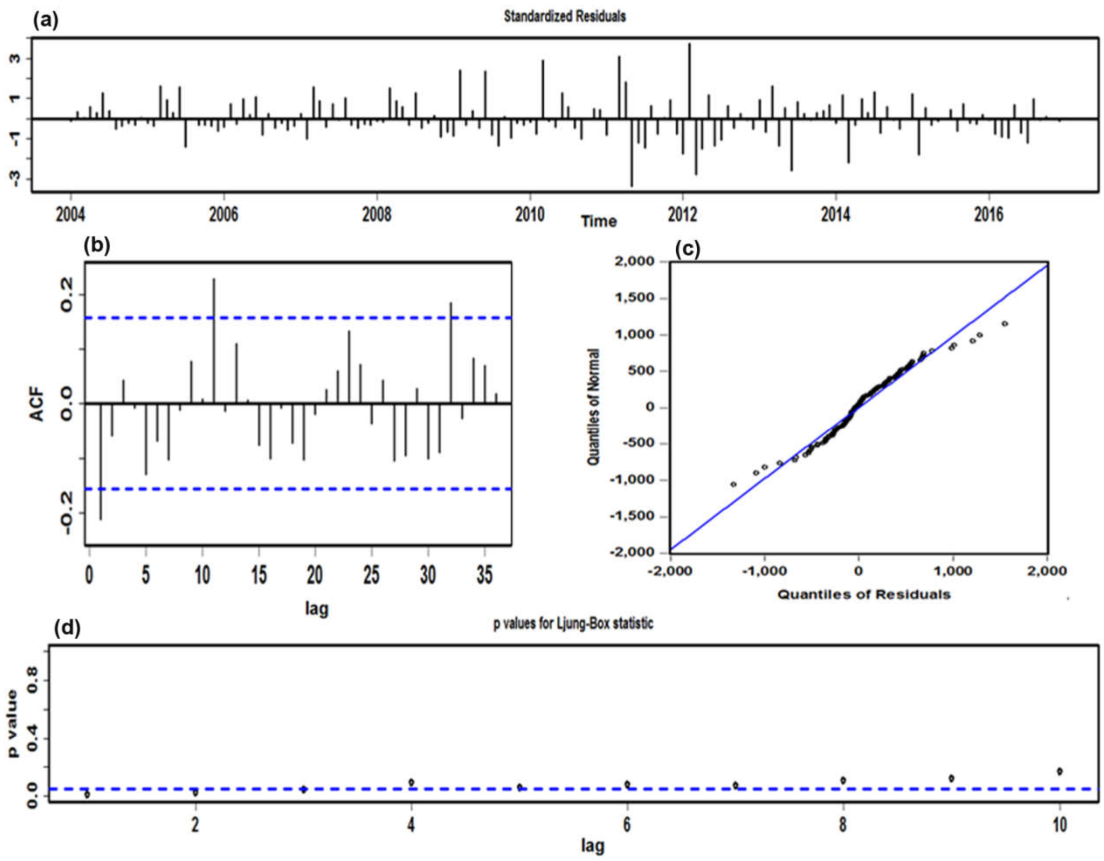

Figure S13. Residual diagnostic plots of the ETS(A,MD,M) model for monthly human brucellosis incidence cases series in mainland China from January 2004 to December 2016. (**a**) Standardized residuals. (**b**) Autocorrelation function(ACF) plot of the time-series residuals. (**c**) Normal Q-Q plot for determining the normality of the time-series residuals. (**d**) *P* values for Ljung-Box statistic. From the graphs above we can see that the residuals are close to be normal, the autocorrelation can be observed at lags 1, 11, and 32, and the *P* values are large except for lags 1 and 2, suggesting that the elected ETS model seemingly can be enhanced.

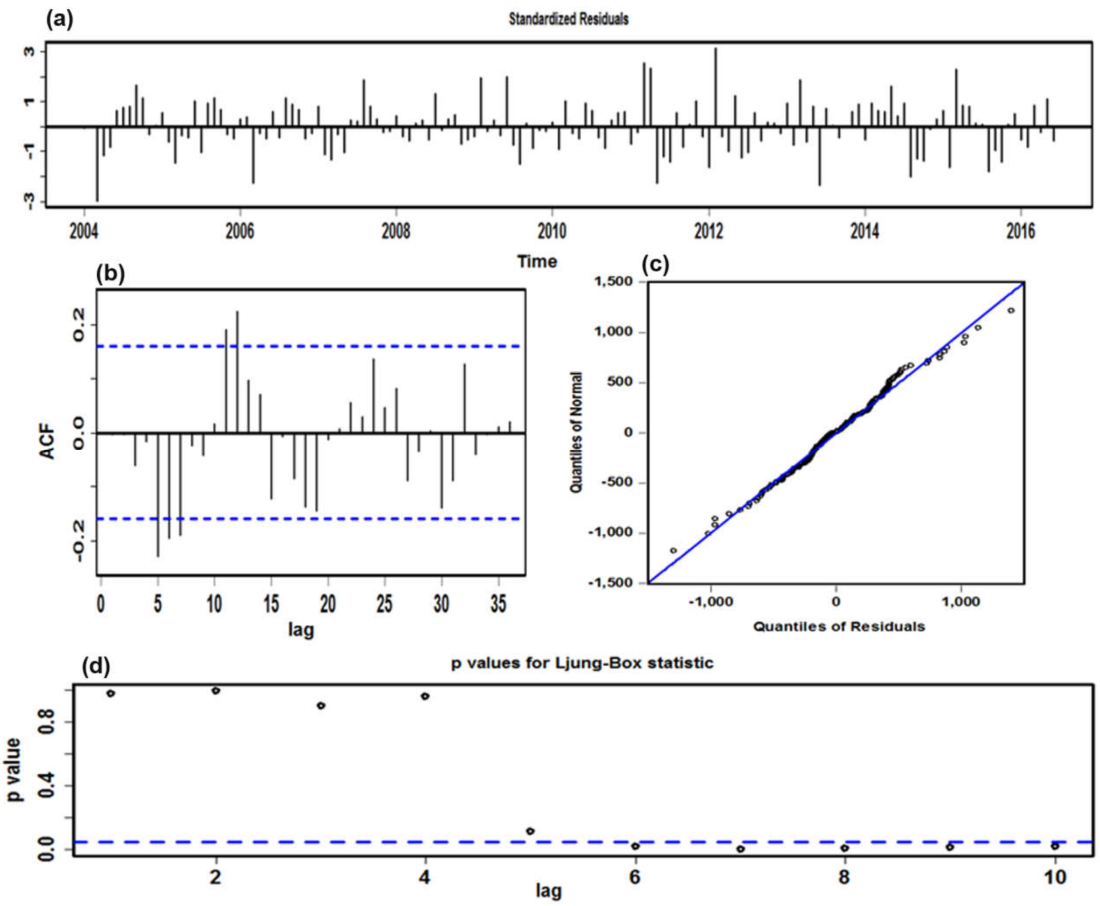

Figure S14. Residual diagnostic plots of the ETS(A,N,A) model for monthly human brucellosis incidence cases series in mainland China from January 2004 to June 2016. (**a**) Standardized residuals. (**b**) Autocorrelation function(ACF) plot of the time-series residuals. (**c**) Normal Q-Q plot for determining the normality of the time-series residuals. (**d**) *P* values for Ljung-Box statistic. As shown in figures above, which indicate that it may be possible to improve the prediction.

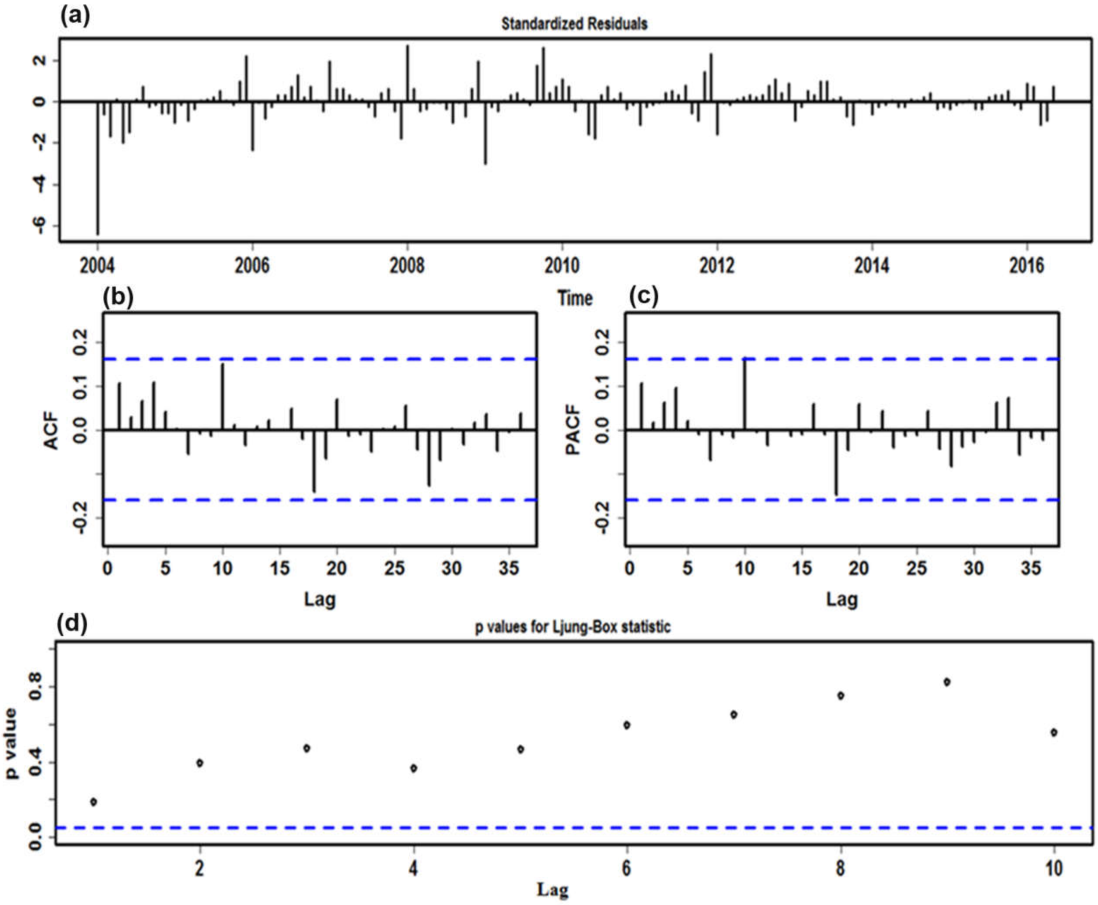

Figure S15. Residual diagnostic plots of the ARIMA(0,1,2)×$(0,1,0)_{12}$ model for approximation generated by coif1 technique from January 2004 to June 2017. (**a**) Standardized residuals. (**b**) Autocorrelation function(ACF) plot of the time-series residuals. (**c**) Partial autocorrelation function(PACF) plot of the time-series residuals. (**d**) *P* values for Ljung-Box statistic. All correlations fall within the estimated 95% uncertainty limits and a large *P* value for Ljung-Box statistic is observed demonstrating that the model is appropriate.

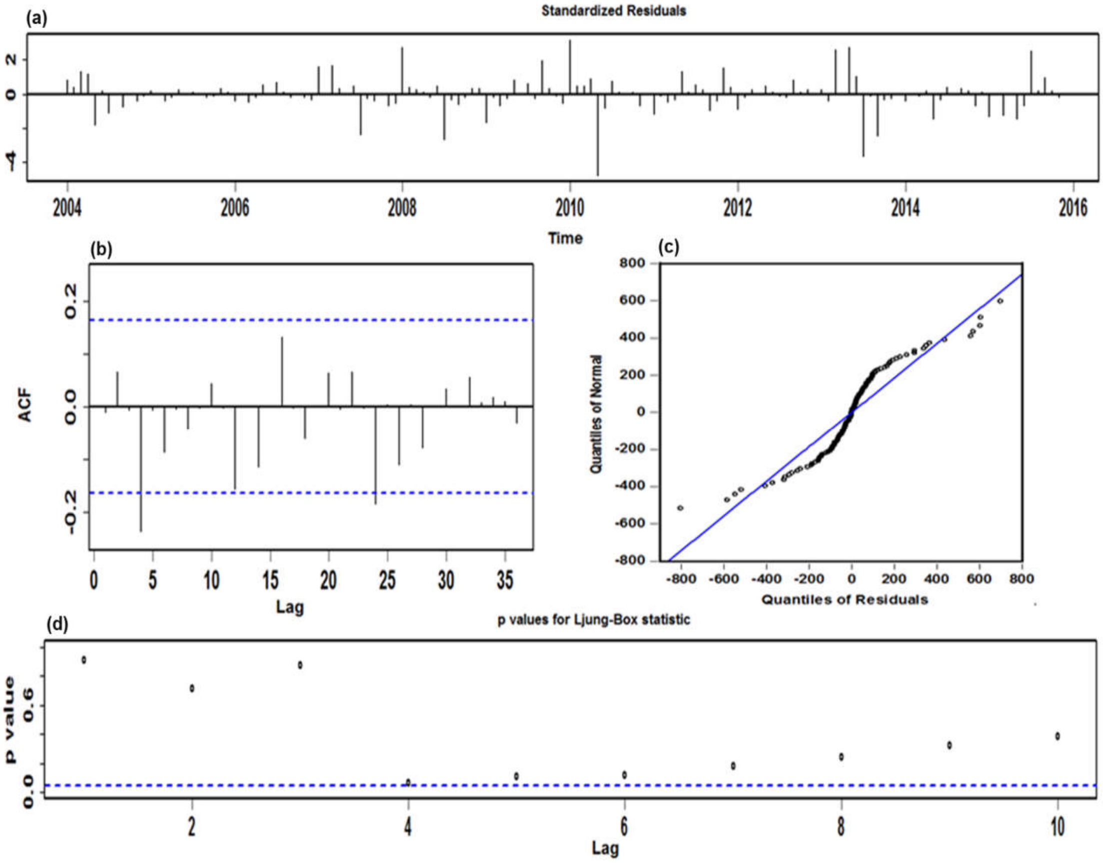

Figure S16. Residual diagnostic plots of the ARIMA(0,1,2)×(0,1,0)$_{12}$ model for approximation generated by coif1 technique from January 2004 to December 2016. (**a**) Standardized residuals. (**b**) Autocorrelation function(ACF) plot of the time-series residuals. (**c**) Normal Q-Q plot for determining the normality of the time-series residuals. (**d**) *P* values for Ljung-Box statistic. There is not significant correlation in the prediction errors and the results for Ljung-Box statistic look good (more than 0.05), indicating that this ARIMA model seems to simulate the time series well.

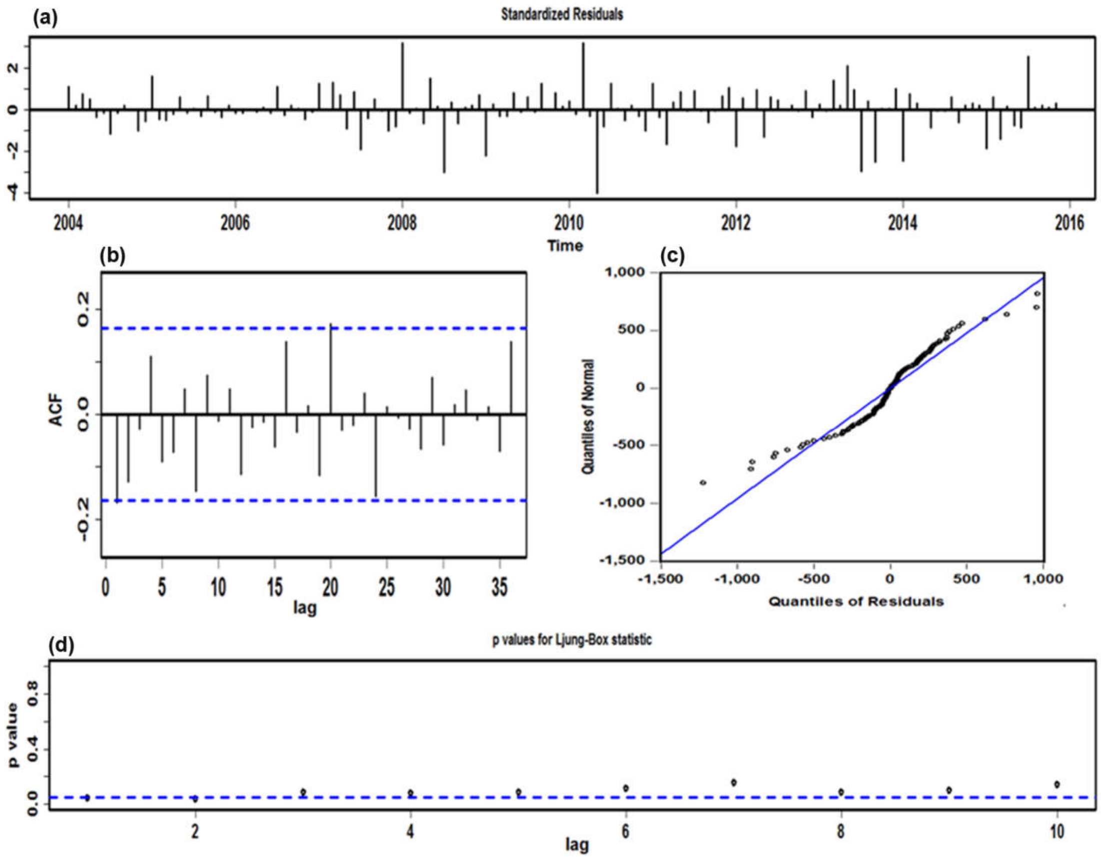

Figure S17. Residual diagnostic plots of the hybrid ARIMA-ETS model for monthly human brucellosis incidence cases series in mainland China from January 2004 to December 2016. (**a**) Standardized residuals. (**b**) Autocorrelation function(ACF) plot of the time-series residuals. (**c**) Normal Q-Q plot for determining the normality of the time-series residuals. (**d**) *P* values for Ljung-Box statistic. The error correlations at lags were approximately independent and the *P* values for Ljung-Box statistic are more than 0.05 apart from the values at lags 1 and 2. It seems that this hybrid method can be employed to fit the data.

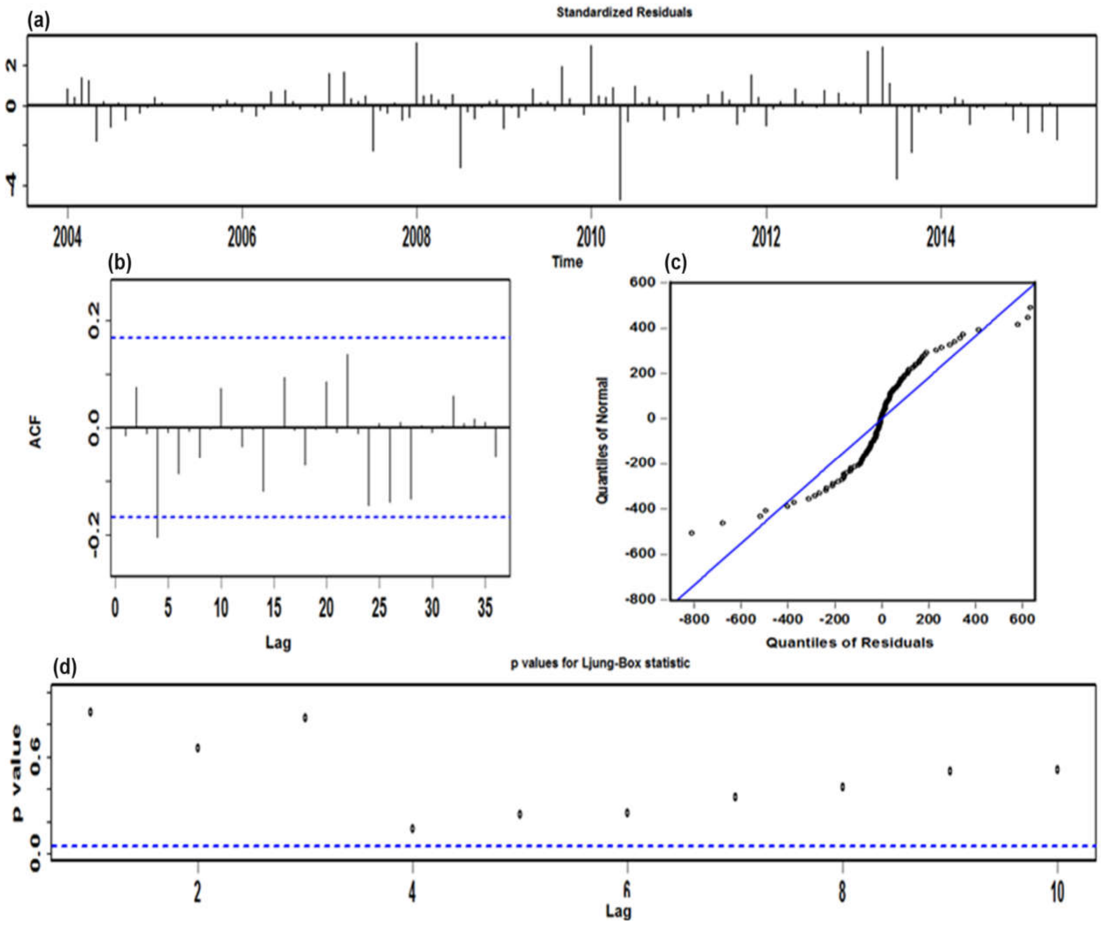

Figure S18. Residual diagnostic plots of the ARIMA(0,1,2)×(1,1,0)$_{12}$ model for approximation generated by coif1 technique from January 2004 to June 2016. (**a**) Standardized residuals. (**b**) Autocorrelation function(ACF) plot of the time-series residuals. (**c**) Normal Q-Q plot for determining the normality of the time-series residuals. (**d**) *P* values for Ljung-Box statistic. The ACF of the residuals shows no apparent departure from the model assumptions, and the Q-statistic is never significant at the lags, suggesting that this is a suitable model for the time series.

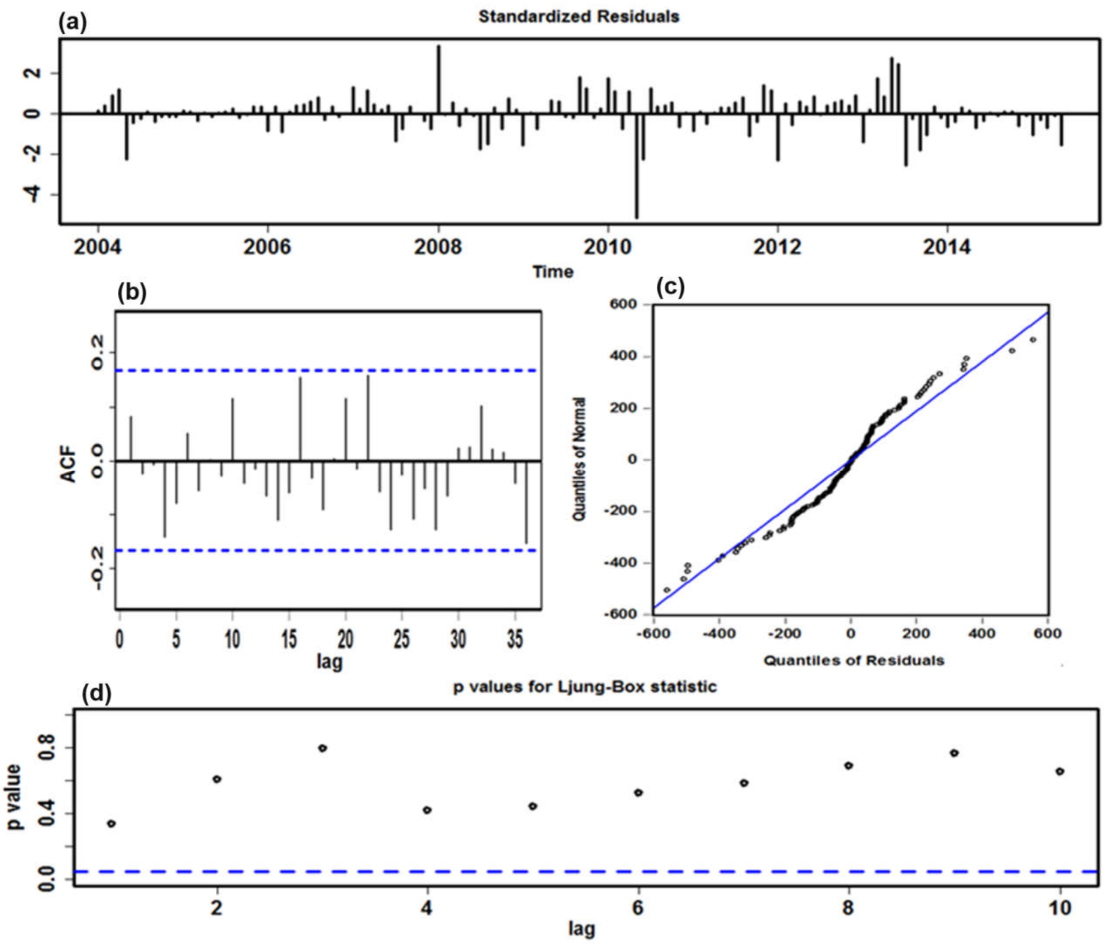

Figure S19. Residual diagnostic plots of the hybrid ARIMA-ETS model for monthly human brucellosis incidence cases series in mainland China from January 2004 to June 2016. (**a**) Standardized residuals. (**b**) Autocorrelation function(ACF) plot of the time-series residuals. (**c**) Normal Q-Q plot for determining the normality of the time-series residuals. (**d**) *P* values for Ljung-Box statistic. It can be seen from the plots above that this hybrid model appears to fit well except for the fact that a distribution with heavy tails than the normal distribution should be employed.

Models	Estimated parameter coefficients of candidate models						Performance indexes of candidate models			
	AR1 (SE)	MA1 (SE)	MA2 (SE)	SAR1 (SE)	SMA1 (SE)	SMA2 (SE)	AIC	AICc	BIC	Log likelihood
$ARIMA(1,1,1)(0,1,1)_{12}$	0.668	-0.869			-0.617		2258.90	2259.19	2270.93	-1125.46
	0.128	0.090			0.078					
$ARIMA(0,1,1)(0,1,1)_{12}$		-0.197			-0.596		2262.30	2262.48	2271.32	-1128.16
		0.095			0.080					
$ARIMA(0,1,0)(0,1,1)_{12}$					-0.648		2264.50	2264.6	2270.52	-1130.26
					0.072					
$ARIMA(0,1,1)(2,1,0)_{12}$		-0.242		-0.500		-0.248	2268.90	2269.22	2280.96	-1130.47
		0.093		0.090		0.082				
$ARIMA(0,1,2)(0,1,1)_{12}$		-0.194	-0.116		-0.590					
		0.087	0.096		0.080					
$ARIMA(1,1,0)(0,1,1)_{12}$	-0.154				-0.611		2263.20	2263.37	2272.21	-1128.60
	0.084				0.077					

Note: when significance of estimated parameter coefficients is around 5% level, SPSS17.0 is used to further diagnose. SE stands for standard error.

Table S1. The estimated parameters and performance indexes of selected candidate models based on original observations from January 2004 to June 2017.

Models	Estimated parameter coefficients of candidate models								Performance indexes of candidate models			
	AR1	AR2	MA1	MA2	SAR1	SAR2	SMA1	SMA2	AIC	AICc	BIC	Log likelihood
	(SE)	(SE)	(SE)	(SE)	(SE)	(SE)	(SE)	(SE)				
ARIMA(1,0,0)(0,1,1)$_{12}$	0.748						-0.615		2165.40	2165.69	2177.28	-1078.70
	0.056						0.068					
ARIMA(1,0,0)(0,1,0)$_{12}$	0.745								2207.37	2207.45	2213.31	-1101.68
	0.055											
ARIMA(1,0,0)(0,1,2)$_{12}$	0.868						-0.594	0.041	2175.02	2175.31	2186.9	-1083.51
	0.046						0.093	0.082				
ARIMA(1,0,1)(1,1,0)$_{12}$	0.875		-0.182		-0.408				2181.92	2182.21	2193.8	-1086.96
	0.049		0.104		0.078							
ARIMA(1,0,0)(2,1,0)$_{12}$	0.837				-0.534	-0.215			2178.14	2178.43	2190.02	-1085.07
	0.047				0.085	0.082						
ARIMA(1,0,2)(1,1,1)$_{12}$	0.916		-0.171	-0.053	-0.039		-0.524		2176.10	2176.71	2193.92	-1082.05
	0.044		0.097	0.101	0.0139		0.119					

Note: when significance of estimated parameter coefficients is around 5% level, SPSS17.0 is used to further diagnose. SE stands for standard error.

Table S2. The estimated parameters and performance indexes of the selected candidate models based on the original observations from January 2004 to December 2016.

Lags	Residuals of ARIMA model		Residuals of ETS model		Residuals of hybrid model	
	Box-Ljung Q	*P*	Box-Ljung Q	*P*	Box-Ljung Q	*P*
1	0.138	0.710	7.068	0.008	4.099	0.043
3	1.043	0.791	7.939	0.047	6.570	0.087
6	2.598	0.857	11.428	0.076	10.355	0.110
9	3.413	0.946	14.151	0.117	14.775	0.097
12	10.525	0.570	22.994	0.028	17.221	0.141
15	13.304	0.579	26.069	0.037	17.933	0.266
18	15.831	0.604	28.771	0.051	21.342	0.263
21	18.507	0.617	30.873	0.076	28.733	0.121
24	19.049	0.749	35.707	0.059	33.275	0.098
27	22.670	0.703	38.369	0.072	33.462	0.182
30	26.995	0.624	42.195	0.069	35.719	0.217
33	34.190	0.410	50.673	0.025	36.215	0.321
36	35.203	0.506	53.100	0.033	40.871	0.265

Table S3. Ljung-Box Q test of the time-series residuals from January 2004 to December 2016 for each of the selected optimal models.

Lags	Observed values		ARIMA model		ETS model		Hybrid model	
	LM-test	*P*	LM-test	*P*	LM-test	*P*	LM-test	*P*
1	118.250	<0.001	13.235	<0.001	9.604	0.001	1.255	0.263
3	131.460	<0.001	13.525	0.004	11.595	0.009	14.333	0.002
6	129.350	<0.001	16.437	0.012	13.189	0.040	22.790	<0.001
9	130.550	<0.001	16.300	0.061	24.857	0.003	26.431	0.002
12	131.880	<0.001	18.972	0.090	30.162	0.003	27.104	0.007
15	130.590	<0.001	28.067	0.021	31.194	0.008	27.268	0.027
18	128.170	<0.001	27.935	0.063	37.224	0.005	26.741	0.084
21	125.480	<0.001	28.583	0.124	38.213	0.012	28.683	0.122
24	122.630	<0.001	34.398	0.078	40.891	0.017	29.051	0.218
27	119.730	<0.001	35.918	0.117	44.122	0.020	28.875	0.367
30	116.920	<0.001	36.317	0.198	44.332	0.044	30.855	0.423
33	114.320	<0.001	36.603	0.305	43.903	0.097	31.094	0.562
36	112.680	<0.001	38.023	0.377	45.466	0.134	38.850	0.343

Table S4. ARCH LM-test of the time-series residuals from January 2004 to December 2016 for each of the selected optimal models.

Models	Estimated parameter coefficients of candidate models								Performance indexes of candidate models			
	AR1 (SE)	AR2 (SE)	MA1 (SE)	MA2 (SE)	SAR1 (SE)	SAR2 (SE)	SMA1 (SE)	SMA2 (SE)	AIC	AICc	BIC	Log likelihood
ARIMA(1,0,0)(0,1,1)$_{12}$	0.715						-0.598		2074.87	2075.17	2086.58	-1033.44
	0.061						0.070					
ARIMA(1,0,0)(1,1,1)$_{12}$	0.863				-0.103		-0.472		2085.8	2086.1	2097.51	-1038.90
	0.047				0.143		0.135					
ARIMA(1,0,1)(0,1,1)$_{12}$	0.903		-0.169				-0.525		2083.74	2084.04	2095.45	-1037.87
	0.043		0.103				0.078					
ARIMA(1,0,2)(0,1,2)$_{12}$	0.913		-0.165	-0.059			-0.553	0.048	2087.12	2087.76	2104.68	-1037.56
	0.047		0.100	0.103			0.093	0.083				
ARIMA(1,0,0)(2,1,0)$_{12}$	0.839				-0.534	-0.196			2087.48	2087.78	2099.19	-1039.74
	0.048				0.085	0.083						
ARIMA(2,0,2)(0,1,1)$_{12}$	0.706	0.188	0.036	-0.087			-0.524		2087.42	2088.07	2104.99	-1037.71
	1.040	0.934	1.036	0.174			0.077					

Note: when significance of estimated parameter coefficients is around 5% level, SPSS17.0 is used to further diagnose. SE stands for standard error.

Table S5. The estimated parameters and performance indexes of the selected candidate models based on the original observations from January 2004 to June 2016

Lags	Residuals of ARIMA model		Residuals of ETS model		Residuals of hybrid model	
	Box-Ljung Q	*P*	Box-Ljung Q	*P*	Box-Ljung Q	*P*
1	0.017	0.898	0.001	0.980	0.921	0.337
3	0.744	0.863	0.583	0.900	1.007	0.800
6	3.088	0.798	14.794	0.022	5.147	0.525
9	4.139	0.902	20.833	0.013	5.729	0.767
12	10.110	0.606	35.055	<0.001	7.998	0.785
15	12.569	0.636	39.971	<0.001	11.132	0.743
18	14.363	0.705	44.475	<0.001	16.256	0.575
21	17.802	0.661	48.130	0.001	18.404	0.623
24	18.073	0.799	52.127	0.001	25.825	0.362
27	21.103	0.781	55.181	0.001	28.402	0.390
30	25.110	0.720	59.086	0.001	32.112	0.362
33	31.344	0.550	63.988	0.001	34.162	0.412
36	32.469	0.637	64.097	0.003	38.887	0.341

Table S6. Ljung-Box Q test of the time-series residuals from January 2004 to June 2016 for each of the selected optimal models.

Lags	Observed values		ARIMA model		ETS model		Hybrid model	
	LM-test	*P*	LM-test	*P*	LM-test	*P*	LM-test	*P*
1	114.150	<0.001	13.752	<0.001	2.095	0.480	4.235	0.040
3	126.300	<0.001	13.661	0.003	3.363	0.339	4.473	0.215
6	124.490	<0.001	17.385	0.015	4.491	0.611	5.702	0.457
9	125.630	<0.001	17.284	0.044	5.629	0.776	6.324	0.707
12	126.720	<0.001	19.869	0.070	8.197	0.770	6.924	0.863
15	125.450	<0.001	26.601	0.032	10.358	0.780	7.809	0.931
18	122.930	<0.001	26.467	0.090	11.987	0.848	8.552	0.969
21	120.200	<0.001	27.013	0.170	15.092	0.818	10.043	0.978
24	117.440	<0.001	35.472	0.062	17.006	0.848	10.734	0.991
27	114.650	<0.001	37.639	0.084	18.603	0.884	11.546	0.996
30	111.850	<0.001	38.760	0.131	20.579	0.901	17.906	0.960
33	109.290	<0.001	38.438	0.237	22.350	0.920	18.262	0.982
36	107.480	<0.001	39.793	0.305	24.130	0.935	20.647	0.981

Table S7. ARCH LM-test of the time-series residuals from January 2004 to June 2016 for each of the selected optimal models.

Models	Compact LL	Likelihood	AIC	BIC	HQ	AMSE
A,N,A	-1397.564	-1215.337	2823.129	2866.355	2840.679	385599.410
A,A,A*	-1397.685	-1215.457	2827.369	2876.771	2847.427	387582.038
A,AD,A	-1397.564	-1215.337	2829.129	2881.618	2850.440	1.00E+100
A,M,A*	-1400.053	-1217.825	2832.105	2881.507	2852.163	370071.521
A,MD,A*	-1412.407	-1230.179	2858.813	2911.302	2880.124	485557.901
M,MD,A*	-1425.098	-1242.871	2884.197	2936.686	2905.508	594558.208
M,A,A	-1471.183	-1288.956	2974.366	3023.768	2994.424	2983455.686
M,AD,A*	-1471.183	-1288.956	2976.366	3028.856	2997.678	2983386.967
M,N,A	-1476.950	-1294.722	2981.899	3025.126	2999.450	1168764.361
A,MD,N*	-1498.641	-1316.414	3007.282	3022.720	3013.550	2455870.682
A,N,N	-1517.043	-1334.816	3038.087	3044.262	3040.594	2540106.319
A,M,N	-1516.204	-1333.977	3040.408	3052.759	3045.423	2437613.481
A,A,N	-1516.949	-1334.721	3041.897	3054.248	3046.912	2536535.243
A,AD,N*	-1516.848	-1334.621	3043.696	3059.134	3049.964	1.00E+100
M,AD,N	-1534.659	-1352.432	3079.318	3094.756	3085.586	2992235.965
M,A,N	-1541.544	-1359.316	3091.087	3103.437	3096.101	2974230.648
M,M,A*	-1536.428	-1354.200	3104.855	3154.257	3124.913	1009427.765
M,MD,N	-1560.567	-1378.340	3131.134	3146.572	3137.403	3645505.911
M,M,N	-1566.600	-1384.372	3141.199	3153.550	3146.214	5408276.110
M,N,N	-1570.288	-1388.060	3144.575	3150.751	3147.083	2619211.969
M,N,M*	-1805.553	-1623.325	3639.105	3682.332	3656.656	104554245.600
A,N,M*	-1868.983	-1686.755	3765.965	3809.192	3783.516	97267412.960
A,A,M*	-2007.668	-1825.441	4047.336	4096.738	4067.394	639201952.800
M,A,M*	-2088.985	-1906.758	4209.970	4259.371	4230.027	5597800424.000
M,AD,M*	-2133.398	-1951.171	4300.797	4353.286	4322.108	1.00E+100
A,AD,M*	-2289.439	-2107.212	4612.878	4665.367	4634.189	1.00E+100
M,M,M*	-3075.144	-2892.917	6182.288	6231.689	6202.346	4.92E+26
A,M,M*	-5188.991	-5006.764	10409.982	10459.384	10430.040	4.92E+26
A,MD,M*	1.00E+100	1.00E+100	1.00E+100	1.00E+100	1.00E+100	1.00E+100
M,MD,M*	1.00E+100	1.00E+100	1.00E+100	1.00E+100	1.00E+100	1.00E+100

Note:*18 models failed to converge.

Table S8. Comparison results of in-sample goodness of fit for the candidate ETS specifications.

Parameters	Values
α	0.956
γ	1e-08
Initial level	1800.783
Initial state 1	-1219.429
Initial state 2	-1271.303
Initial state 3	-1405.407
Initial state 4	-558.127
Initial state 5	832.538
Initial state 6	1747.280
Initial state 7	2036.841
Initial state 8	1688.887
Initial state 9	1028.575
Initial state 10	212.264
Initial state 11	-1430.690
Initial state 12	-1661.429

Table S9. Initial parameters of ETS(A,N,A) model.

Models	Compact LL	Likelihood	AIC*	BIC	HQ	AMSE
A,MD,M*	-1331.124	-1158.589	2696.248	2748.095	2717.306	334794.461
A,N,A	-1341.661	-1169.127	2711.323	2754.021	2728.665	385586.122
A,AD,A*	-1339.931	-1167.396	2713.861	2765.709	2734.919	1E+100
A,A,A*	-1341.572	-1169.037	2715.144	2763.941	2734.963	386163.6501
A,M,A*	-1353.622	-1181.088	2739.244	2788.041	2759.063	400387.4556
A,MD,A*	-1352.852	-1180.317	2739.703	2791.551	2760.762	438903.9937
M,MD,A*	-1373.274	-1200.740	2780.548	2832.396	2801.606	555047.5583
M,A,A*	-1411.841	-1239.307	2855.682	2904.480	2875.502	2987980.252
M,AD,A	-1411.841	-1239.307	2857.682	2909.530	2878.740	2987886.917
M,N,A	-1417.620	-1245.085	2863.239	2905.937	2880.582	1136280.587
A,AD,N	-1437.689	-1265.154	2885.378	2900.627	2891.571	1E+100
A,MD,N*	-1440.386	-1267.852	2890.772	2906.022	2896.966	2508614.963
A,N,N	-1458.565	-1286.030	2921.129	2927.229	2923.607	2586968.544
A,M,N	-1457.438	-1284.904	2922.876	2935.076	2927.831	2458241.711
A,A,N	-1458.539	-1286.004	2925.077	2937.277	2930.032	2585857.728
M,AD,N	-1475.276	-1302.742	2960.553	2975.802	2966.746	3069217.838
M,A,N	-1482.044	-1309.510	2972.089	2984.288	2977.044	3049846.853
M,M,A*	-1475.878	-1303.344	2983.756	3032.554	3003.576	1923480.504
M,MD,N	-1500.400	-1327.866	3010.800	3026.049	3016.994	3673734.128
M,M,N	-1506.420	-1333.885	3020.839	3033.039	3025.794	5530971.100
M,N,N	-1509.497	-1336.962	3022.993	3029.093	3025.471	2662964.027
M,MD,M	-1534.686	-1362.152	3103.372	3155.220	3124.430	149866454.500
M,N,M*	-1920.815	-1748.281	3869.630	3912.328	3886.972	705695689.200
M,AD,M*	-1957.696	-1785.161	3949.391	4001.239	3970.449	1E+100
A,AD,M*	-1966.727	-1794.193	3967.454	4019.302	3988.513	1E+100
A,A,M*	-2159.983	-1987.449	4351.966	4400.764	4371.785	3272582461
A,N,M*	-2222.563	-2050.029	4473.127	4515.825	4490.469	16398980113
M,A,M*	-2480.846	-2308.311	4993.691	5042.489	5013.511	67716774517
A,M,M*	-3130.395	-2957.861	6292.791	6341.588	6312.610	2.07858E+16
M,M,M*	-4750.016	-4577.482	9532.033	9580.831	9551.852	4.62379E+58

Note: *17 models failed to converge

Table S10. Comparison results of in-sample goodness of fit from January 2004 to December 2016 for the candidate ETS specifications.

Parameters	Values
α	1
β	0
γ	0
θ	1
Initial level:	274.463
Initial trend:	0.995
Initial state 1:	0.644
Initial state 2:	0.612
Initial state 3:	0.559
Initial state 4:	0.823
Initial state 5:	1.244
Initial state 6:	1.534
Initial state 7:	1.610
Initial state 8:	1.533
Initial state 9:	1.320
Initial state 10:	1.056
Initial state 11:	0.545
Initial state 12:	0.520

Table S11. Initial parameters of ETS(A,MD,M) model.

Models	Compact LL	Likelihood	AIC	BIC	HQ	AMSE
A,N,A	-1288.792	-1125.835	2605.584	2647.733	2622.708	393920.7875
A,AD,A	-1287.126	-1124.169	2608.252	2659.433	2629.045	1E+100
A,A,A	-1288.643	-1125.686	2609.286	2657.456	2628.856	394143.7443
A,MD,A*	-1288.844	-1125.887	2611.688	2662.869	2632.481	394309.4907
A,M,A*	-1293.691	-1130.734	2619.382	2667.552	2638.952	385926.4427
M,MD,A*	-1312.704	-1149.747	2659.408	2710.589	2680.201	590939.7629
M,M,A*	-1319.741	-1156.784	2671.483	2719.653	2691.053	654738.739
M,A,A*	-1353.831	-1190.874	2739.662	2787.832	2759.232	3416255.029
M,AD,A*	-1353.831	-1190.874	2741.662	2792.842	2762.455	3416326.944
A,AD,N	-1379.871	-1216.914	2769.743	2784.796	2775.858	1E+100
A,MD,N*	-1382.381	-1219.425	2774.763	2789.816	2780.879	2602057.245
A,N,N	-1399.414	-1236.457	2802.829	2808.850	2805.275	2571180.301
A,M,N	-1398.871	-1235.914	2805.741	2817.784	2810.634	2489313.96
A,A,N	-1399.247	-1236.291	2806.495	2818.537	2811.387	2563353.515
M,N,A*	-1396.526	-1233.569	2821.052	2863.201	2838.176	115429534.1
M,AD,N	-1414.633	-1251.676	2839.265	2854.318	2845.381	2981974.907
M,A,N*	-1430.684	-1267.727	2869.369	2881.411	2874.261	15711331.32
M,MD,N	-1439.358	-1276.401	2888.716	2903.770	2894.832	3649848.596
M,M,N	-1444.774	-1281.818	2897.549	2909.591	2902.441	5361492.572
M,N,N	-1447.776	-1284.819	2899.552	2905.573	2901.998	2656348.279
M,N,M*	-1744.657	-1581.700	3517.314	3559.463	3534.438	1505042675
A,N,M*	-1839.086	-1676.129	3706.171	3748.320	3723.295	775303112.2
A,AD,M*	-1844.861	-1681.905	3723.723	3774.904	3744.516	1E+100
M,A,M*	-1863.351	-1700.395	3758.703	3806.873	3778.273	703748014.5
A,A,M*	-1885.911	-1722.954	3803.822	3851.992	3823.392	825512271.5
M,MD,M*	-1900.180	-1737.223	3834.361	3885.541	3855.154	1.81268E+14
M,AD,M*	-1901.492	-1738.535	3836.984	3888.165	3857.777	1E+100
A,MD,M*	-2647.546	-2484.589	5329.092	5380.273	5349.885	1.81268E+14
M,M,M*	-2737.498	-2574.541	5506.995	5555.165	5526.565	4.18576E+36
A,M,M*	-6226.777	-6063.820	12485.553	12533.724	12505.123	9.13191E+34

Note: *19 models failed to converge.

Table S12. Comparison results of in-sample goodness of fit from January 2004 to June 2016 for the candidate ETS specifications.

Parameters	Values
α	0.963
γ	1.00E-04
Initial level	1495.005
Initial state 1	-1279.531
Initial state 2	-1322.092
Initial state 3	-1428.213
Initial state 4	-553.854
Initial state 5	810.566
Initial state 6	1774.292
Initial state 7	2095.35
Initial state 8	1785.007
Initial state 9	1132.772
Initial state 10	195.264
Initial state 11	-1509.874
Initial state 12	-1699.686

Table S13. Initial parameters of ETS(A,N,A) model.

Models	Estimated parameter coefficients of candidate models							Performance indexes of candidate models			
	AR1 (SE)	AR2 (SE)	MA1 (SE)	MA2 (SE)	SAR1 (SE)	SMA1 (SE)	SMA2 (SE)	AIC	AICc	BIC	Log likelihood
ARIMA(0,1,2)(0,1,0)$_{12}$			0.744	-0.256				2058.5	2058.66	2067.51	-1026.25
			0.085	0.083							
ARIMA(0,1,0)(0,1,1)$_{12}$						-0.294		2117.4	2117.48	2123.4	-1056.7
						0.128					
ARIMA(0,1,0)(0,1,2)$_{12}$						-0.245	-0.230	2111.8	2111.99	2120.83	-1052.91
						0.087	0.082				
ARIMA(1,1,0)(0,1,2)$_{12}$	0.365					-0.278	-0.258	2093.3	2093.61	2105.35	-1042.67
	0.078					0.089	0.085				
ARIMA(2,1,0)(0,1,2)$_{12}$	0.463	-0.261				-0.255	-0.230	2086.4	2086.77	2101.37	-1038.18
	0.082	0.086				0.089	0.085				
ARIMA(2,1,0)(0,1,2)$_{12}$	0.464	-0.256			0.160	-0.406	-0.187	2088.2	2088.77	2106.20	-1038.09
	0.082	0.087			0.383	0.378	0.141				

Note: when significance of estimated parameter coefficients is around 5% level, SPSS17.0 is used to further diagnose. SE stands for standard error.

Table S14. The estimated parameters and performance indexes of selected candidate models based on approximation from January 2004 to June 2017.

Models	Compact LL	Likelihood	AIC	BIC	HQ	AMSE
A,N,A	-1270.251	-1088.024	2568.503	2611.729	2586.053	42613.940
A,A,A	-1270.249	-1088.022	2572.498	2621.900	2592.556	42616.923
A,AD,A	-1270.251	-1088.024	2574.503	2626.992	2595.814	1.00E+100
M,A,A	-1340.411	-1158.183	2712.821	2762.223	2732.879	88952.393
A,N,N*	-1357.602	-1175.375	2719.204	2725.379	2721.711	117316.983
A,A,N	-1357.600	-1175.373	2723.200	2735.550	2728.214	117313.252
A,AD,N	-1357.594	-1175.367	2725.188	2740.626	2731.456	1.00E+100
M,N,N	-1372.799	-1190.572	2749.599	2755.774	2752.106	157569.209
M,AD,A*	-1372.550	-1190.323	2779.100	2831.590	2800.412	1.00E+100
M,A,N	-1386.573	-1204.346	2781.147	2793.497	2786.161	202998.483
M,AD,N*	-1386.573	-1204.346	2783.147	2798.585	2789.415	202998.555
M,N,A	-1386.210	-1203.983	2800.420	2843.646	2817.971	62232.360

Note: *3 models failed to converge

Table S15. Comparison results of in-sample goodness of fit for the candidate ETS specifications.

Parameters	Values
α	1e-04
γ	1e-04
Initial level	7.9579
Initial state 1	272.351
Initial state 2	80.587
Initial state 3	-463.708
Initial state 4	127.834
Initial state 5	189.848
Initial state 6	-207.276
Initial state 7	274.723
Initial state 8	-198.232
Initial state 9	170.242
Initial state 10	189.099
Initial state 11	-600.756
Initial state 12	165.289

Table S16. Initial parameters of ETS(A,N,A) model.

Models	Estimated parameter coefficients of candidate models								Performance indexes of candidate models			
	AR1 (SE)	AR2 (SE)	MA1 (SE)	MA2 (SE)	SAR1 (SE)	SAR2 (SE)	SMA1 (SE)	SMA2 (SE)	AIC	AICc	BIC	Log likelihood
ARIMA(0,1,2)(0,1,0)$_{12}$			0.763	-0.237					1961.72	1961.89	1970.6	-977.86
			0.082	0.079								
ARIMA(2,0,0)(0,1,2)$_{12}$	1.312	-0.461					-0.258	-0.242	1992.43	1993.04	2010.25	-990.21
	0.074	0.075					0.085	0.080				
ARIMA(1,0,1)(0,1,1)$_{12}$	0.827		1.000				-0.250		1968.71	1968.99	1980.58	-980.35
	0.047		0.023				0.107					
ARIMA(0,1,1)(0,1,1)$_{12}$			1.000				-0.292		1963.97	1964.14	1972.86	-978.98
			0.023				0.106					
ARIMA(1,0,0)(2,1,0)$_{12}$	0.927				-0.201	-0.219			2025.65	2025.94	2037.53	-1008.83
	0.030				0.086	0.082						
ARIMA(1,0,0)(2,1,1)$_{12}$	0.934				0.186	-0.164	-0.409		2025.88	2026.32	2040.73	-1007.94
	0.029				0.239	0.100	0.233					

Note: when significance of estimated parameter coefficients is around 5% level, SPSS17.0 is used to further diagnose. SE stands for standard error.

Table S17. The estimated parameters and performance indexes of selected candidate models based on approximation from January 2004 to December 2016.

Models	Compact LL	Likelihood	AIC*	BIC	HQ	AMSE
A,N,A	-1218.432	-1045.898	2464.864	2507.562	2482.206	41925.694
A,A,A	-1218.429	-1045.895	2468.859	2517.656	2488.678	41928.893
A,AD,A	-1218.320	-1045.785	2470.640	2522.487	2491.698	1.00E+100
A,N,N	-1306.036	-1133.501	2616.072	2622.171	2618.549	119838.800
A,A,N	-1306.034	-1133.500	2620.069	2632.268	2625.024	119835.851
A,AD,N	-1306.036	-1133.501	2622.072	2637.321	2628.265	1.00E+100
M,A,A	-1299.987	-1127.452	2631.973	2680.771	2651.793	125660.789
M,N,N	-1319.865	-1147.331	2643.731	2649.830	2646.208	161018.947
M,AD,A*	-1318.403	-1145.868	2670.805	2722.653	2691.863	1.00E+100
M,A,N	-1333.390	-1160.856	2674.780	2686.980	2679.735	207343.323
M,AD,N	-1333.390	-1160.856	2676.780	2692.030	2682.974	1.00E+100
M,N,A	-1330.239	-1157.705	2688.478	2731.176	2705.820	60155.956

Note:*1 model failed to converge

Table S18. Comparison results of goodness of fit for the candidate ETS specifications based on detail from January 2004 to December 2016.

Parameters	Values
α	1.00E-04
γ	3.00E-04
Initial level:	1.339
Initial state 1:	263.477
Initial state 2:	75.880
Initial state 3:	-456.419
Initial state 4:	123.816
Initial state 5:	185.632
Initial state 6:	-207.935
Initial state 7:	271.141
Initial state 8:	-195.028
Initial state 9:	177.615
Initial state 10:	167.780
Initial state 11:	-615.547
Initial state 12:	209.588

Table S19. Initial parameters of ETS(A,N,A) model.

Models	Estimated parameter coefficients of candidate models								Performance indexes of candidate models			
	AR1 (SE)	AR2 (SE)	MA1 (SE)	MA2 (SE)	SAR1 (SE)	SAR2 (SE)	SMA1 (SE)	SMA2 (SE)	AIC	AICc	BIC	Log likelihood
ARIMA(0,1,2)(1,1,0)$_{12}$			0.764	-0.237	-0.176				1875.81	1876.12	1887.49	-933.91
			0.089	0.081	0.085							
ARIMA(2,0,0)(1,1,0)$_{12}$	1.335	-0.438			-0.159				1916.22	1916.52	1927.93	-954.11
	0.077	0.077			0.085							
ARIMA(0,1,1)(1,1,0)$_{12}$			0.994		-0.176				1882.12	1882.30	1890.88	-938.06
			0.075		0.085							
ARIMA(0,1,1)(1,1,1)$_{12}$			1.000		0.300		-0.533		1881.10	1881.40	1892.78	-936.55
			0.090		0.222		0.197					
ARIMA(0,1,1)(0,1,2)$_{12}$			1.000				-0.220	-0.120	1880.58	1880.88	1892.26	-936.29
			0.057				0.087	0.084				
ARIMA(1,0,0)(2,1,1)$_{12}$	0.937				0.221	-0.081	-0.446		1943.01	1943.47	1957.65	-966.51
	0.030				0.293	0.116	0.289					

Note: when significance of estimated parameter coefficients is around 5% level, SPSS17.0 is used to further diagnose. SE stands for standard error.

Table S20. The estimated parameters and performance indexes of selected candidate models based on approximation from January 2004 to June 2016.

Models	Compact LL	Likelihood	AIC	BIC	HQ	AMSE
A,N,A*	-1170.225	-1007.268	2368.450	2410.598	2385.573	42983.553
A,A,A	-1170.221	-1007.264	2372.442	2420.612	2392.012	42986.952
A,AD,A	-1170.225	-1007.268	2374.450	2425.630	2395.243	1E+100
M,A,A	-1234.744	-1071.788	2501.489	2549.659	2521.059	96017.522
A,N,N	-1252.468	-1089.511	2508.936	2514.957	2511.382	119208.976
A,A,N	-1252.467	-1089.510	2512.933	2524.976	2517.826	119206.143
A,AD,N	-1252.467	-1089.510	2514.933	2529.986	2521.049	119206.142
M,N,N	-1263.413	-1100.457	2530.827	2536.848	2533.273	160296.790
M,A,N	-1276.577	-1113.620	2561.154	2573.197	2566.047	206886.038
M,N,A	-1267.170	-1104.213	2562.340	2604.489	2579.464	62078.594
M,AD,N*	-1276.578	-1113.621	2563.155	2578.208	2569.271	1E+100
M,AD,A	-1265.531	-1102.574	2565.061	2616.242	2585.854	1E+100

Note: *2 models failed to converge

Table S21. Comparison results of goodness of fit for the candidate ETS specifications based on detail from January 2004 to June 2016.

Parameters	Values
α	2.00E-04
γ	1.00E-04
Initial level	1.222645
Initial state 1	253.1241
Initial state 2	76.61696
Initial state 3	-442.7663
Initial state 4	125.2954
Initial state 5	168.7994
Initial state 6	-197.3144
Initial state 7	271.2573
Initial state 8	-194.9125
Initial state 9	177.7308
Initial state 10	167.8967
Initial state 11	-615.4313
Initial state 12	209.704

Table S22. Initial parameters of ETS(A,N,A) model.
